# A DFT Study on the Mechanism of Selective Formation of Substituted Azepines From 1‐Azabutadienes and Cyclopropanes

**DOI:** 10.1002/chem.70960

**Published:** 2026-04-03

**Authors:** Ryo Kobayashi, Chao Wang, Shuto Kosuge, Yuji Matsuya, Kenji Sugimoto, Keiichi Hirano

**Affiliations:** ^1^ Institute of Medical Pharmaceutical, and Health Sciences Kanazawa University Kanazawa Japan; ^2^ Graduate School of Pharmaceutical Sciences Tohoku University Sendai Japan; ^3^ Graduate School of Medicine and Pharmaceutical Sciences University of Toyama Toyama Japan; ^4^ Department of Biomolecular Chemistry Faculty of Science and Technology Kyoto Prefectural University Kyoto Japan

**Keywords:** azepine, DFT calculation, Lewis acid, orthogonal relay catalysis, reaction mechanism

## Abstract

Density Functional Theory (DFT) calculations were performed to elucidate the reaction mechanism of the Mg‐catalyzed cyclization between an azadiene‐type imine and a cyclopropane. The results reveal that the [4+3] cyclization pathway leading to azepine formation is both kinetically and thermodynamically preferred over the intuitively more accessible [3+2] pathway toward pyrrolidine formation. This unusual selectivity arises from the interplay of opposing electronic and steric effects, with steric effects ultimately dominating.

## Graphical abstract

The origin of the unusual regioselectivity in the Mg‐catalyzed cyclization of azadiene‐type imines with cyclopropanes is elucidated by DFT calculations. Although five‐membered rings are typically favored, energy decomposition analysis reveals that severe steric congestion induces a highly distorted transition state, rendering the five‐membered pathway kinetically inaccessible and leading to selective formation of seven‐membered azepine products.

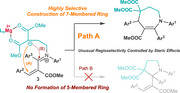

## 1 Introduction

**FIGURE 1:**
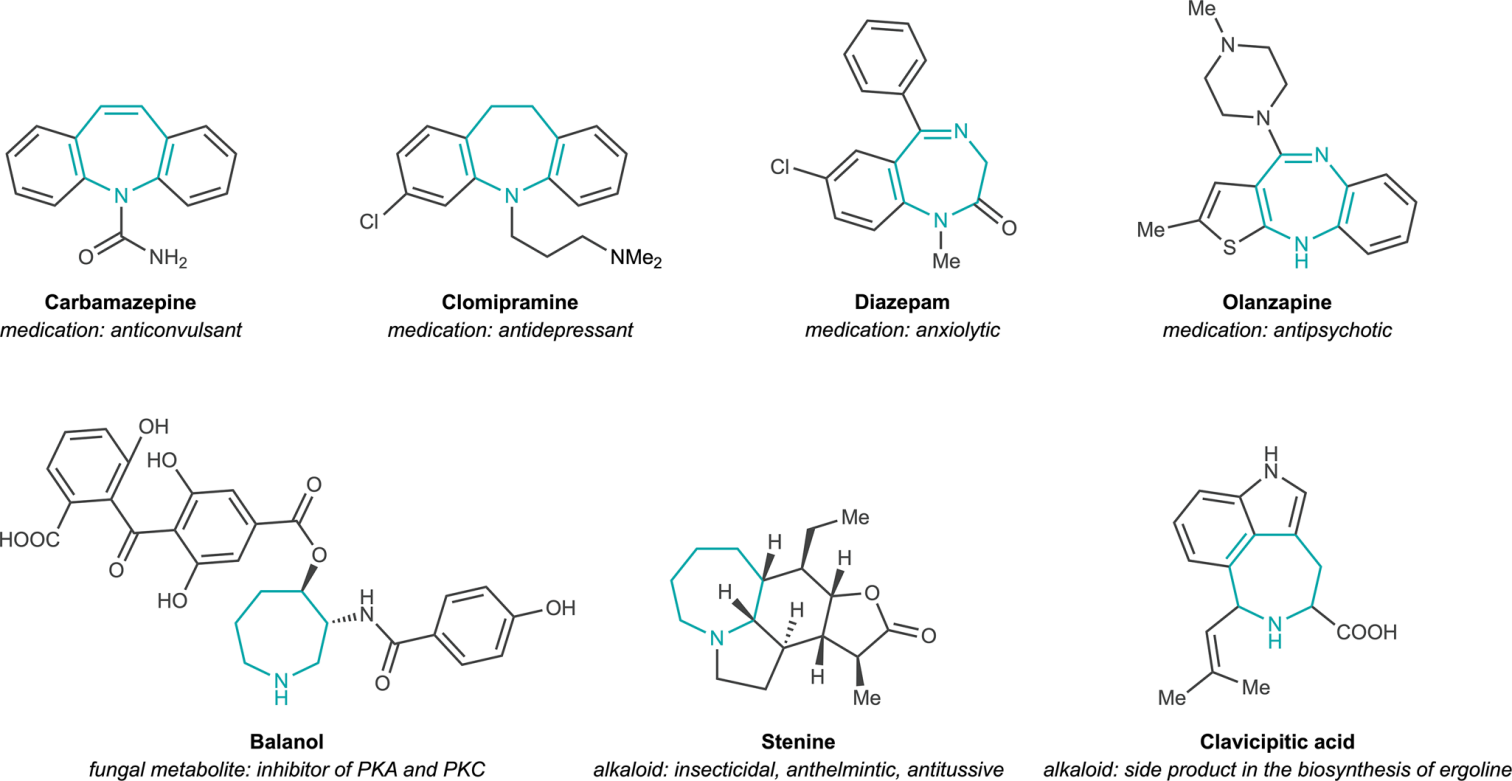
Representative natural products and pharmaceutical molecules containing azepine skeletons.

**SCHEME 1:**
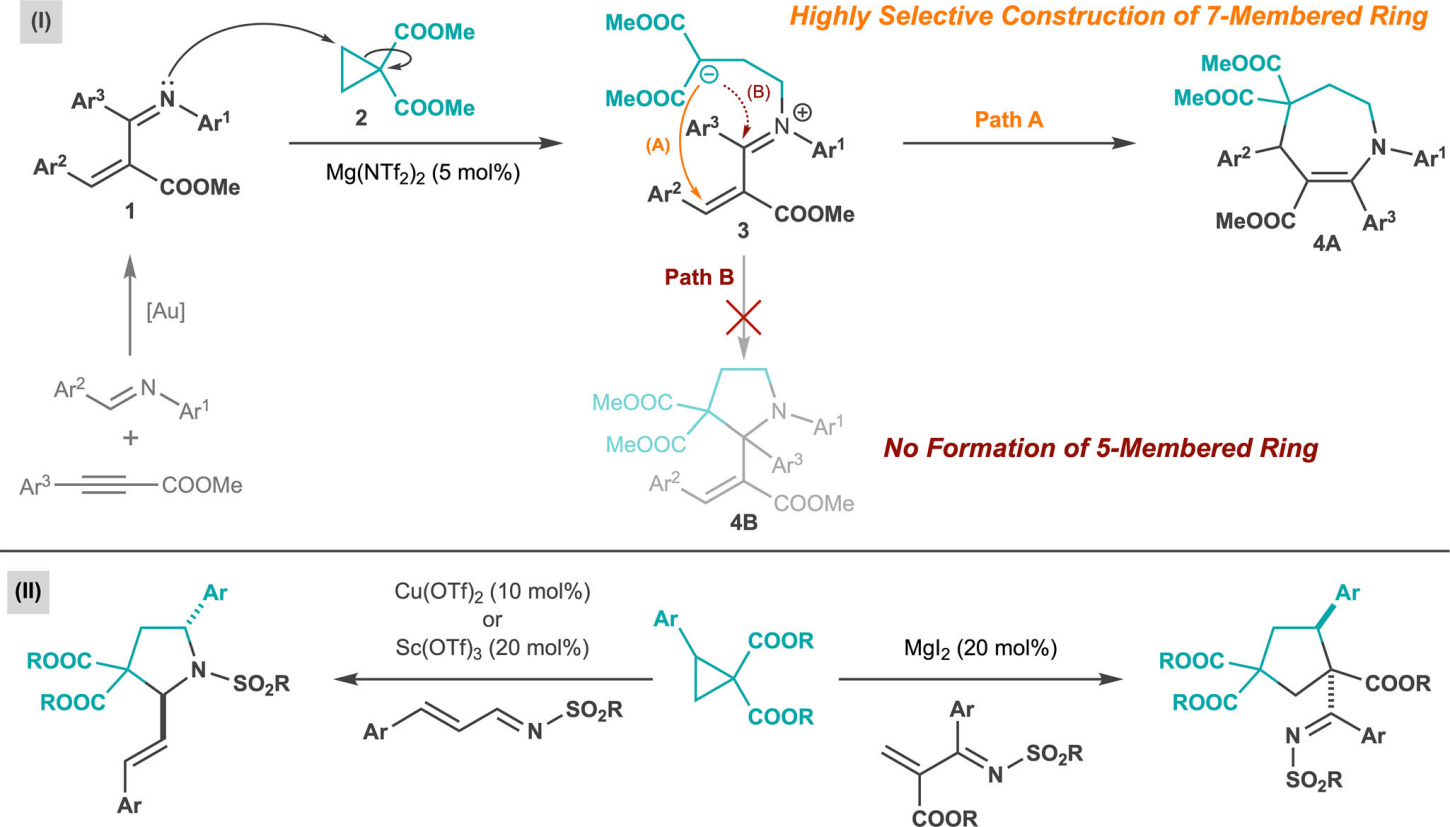
Selected examples for Lewis‐acid‐catalyzed cycloaddition reactions between 1‐azabutadienes and cyclopropanes.

The azepine skeleton, as a representative medium‐sized *N*‐heterocycle, is an important structural scaffold found in a wide range of alkaloids. Owing to its valuable biological activities, including anticancer, antimicrobial, and anti‐inflammatory properties, the azepine framework has been recognized as a privileged chemical motif in pharmaceutical molecules (Figure 1) [1, 2, 3, 4]. Consequently, azepine‐containing compounds have attracted considerable attention from medicinal chemists, and development of divergent synthetic methodologies is eagerly anticipated [5, 6, 7, 8, 9, 10, 11, 12]. In 2024 Sugimoto, Matsuya, and coworkers reported a three‐component synthesis of substituted azepines via Au/Mg orthogonal relay catalysis (Scheme 1‐I) [13]. In this transformation, an in situ‐generated azadiene, derived from an aldimine and a propiolate derivative under Au catalysis [14, 15, 16], was converted to the corresponding azepine in the presence of dimethyl cyclopropane‐1,1‐dicarboxylate, and an Mg catalyst. Compared with previous studies, this method is noteworthy in the following respects. First, it is synthetically attractive because it enables efficient construction of azepine skeletons through a concise and operationally simple protocol. Although several impressive precedents for azepine synthesis based on [4+3] cyclization reactions have been reported, these approaches typically require labor‐intensive, multistep preparation of starting materials [17, 18, 19, 20, 21, 22]. In contrast, the present one‐pot strategy allows facile, rapid, and straightforward diversification of azepines. More importantly, this reaction exhibits counterintuitive chemo‐ and regioselectivity. For example, cyclization reactions between similar azadienes and cyclopropanes under Lewis acidic conditions, as reported by Banerjee, selectively deliver five‐membered rings, namely, cyclopentanes or pyrrolidines depending on the choice of metal catalyst, without any indication of seven‐membered ring formation (Scheme 1‐II) [23]. Banerjee's observation is consistent with the general notion regarding intramolecular cyclization selectivity, wherein five‐membered ring formation is favored over seven‐membered ring due to comparatively smaller entropic and enthalpic penalties. However, the origin of the unusual selectivity leading to seven‐membered ring formation observed in the current Au/Mg relay system remained unclear. In this study, we report the mechanistic rationale for azepine formation based on DFT calculations analyzing the reaction pathways leading to both five‐ and seven‐membered ring formation in the Sugimoto–Matsuya system.

## 2 Results and Discussion

DFT calculations were performed with the Gaussian 16 (Revision B.01) program [24] and the GRRM 17 program [25, 26, 27, 28, 29], which is based on Gaussian. Structure optimizations and frequency calculations were carried out at the level of M062X [30, 31]/6‐31G* level of theory. Single‐point energies including solvent effects of 1,4‐dioxane using the SMD solvation model [32] in the SCRF method and empirical dispersion correction (empirical dispersion = GD3) were calculated at the M062X/6‐31++G** level based on the optimized geometries. Gibbs free energies were evaluated by combining the single‐point energies with thermal and zero‐point energy corrections and were subsequently used for discussion. The intrinsic reaction coordinate (IRC) method was used to track minimum energy paths from transition states to the corresponding local minima [33, 34, 35, 36]. Energy decomposition analysis was performed based on the optimized geometries at the same level of theory used for the single‐point energy calculations [37].

**FIGURE 2:**
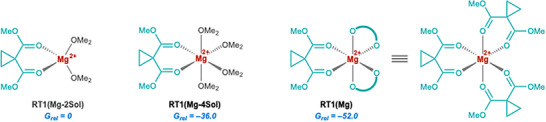
The coordination states of Mg. *G*_rel_: relative Gibbs‐free energy in kcal/mol. Structure optimization and frequency: M062X/6‐31g*. Single‐point energy with consideration of solvent effect and empirical dispersion correction: M062X/6‐311++G** with SCRF = (SMD, solvent = 1,4‐dioxane) and empirical dispersion = GD3.

First, to identify the active species in the Mg‐catalyzed process, we investigated the coordination environment of an Mg center. Because the bis(trifluoromethanesulfonyl)imide anion(NTf_2_^–^) in Mg(NTf_2_)_2_ binds weakly to Mg^2+^ cation, Mg(NTf_2_)_2_ undergoes extensive solvolysis in solution, giving ion pairs consisting of [NTf_2_]^–^ and solvated Mg^2+^ species, particularly in the presence of bidentate ligands [38]. Our calculations on the formation of such ion pairs show that this process is strongly exothermic (see Supporting Information), which is consistent with previous reports [38, 39]. Therefore, to reduce the computational cost, we focused on the cationic part of the system, that is, the coordination of Mg^2+^ with dimethyl cyclopropane‐1,1‐dicarboxylate (**RT1**). On this basis, the three plausible Mg coordination structures were considered for theoretical calculations (Figure 2). The four‐coordinated Mg complex **RT1(Mg‐2sol)** (Δ*G* = 0.0 kcal/mol) is expected to accommodate two additional ethereal solvents to form the six‐coordinated species **RT1(Mg‐4sol)**, a process, that is, highly exergonic (Δ*G* = –36.0 kcal/mol). The complex **RT1(Mg)**, in which Mg^2+^ is chelated by three **RT1** molecules acting as bidentate ligands, was computed to be even more thermodynamically stable than **RT1(Mg‐4sol)** (Δ*G* = –52.0 kcal/mol). We therefore selected the chelated **RT1(Mg)** complex for subsequent DFT investigations of the reaction mechanism.

**SCHEME 2:**
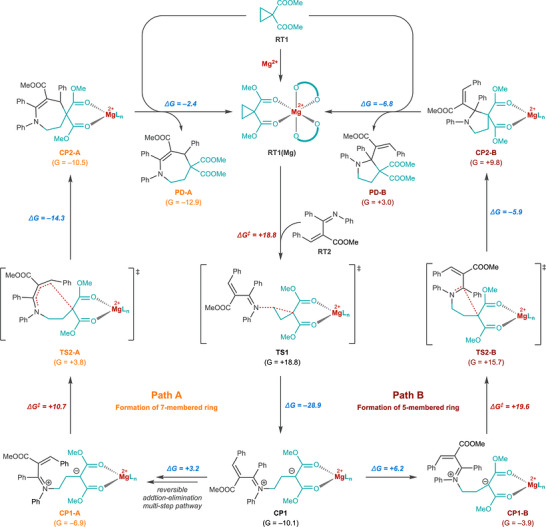
Reaction mechanism for the formation of seven‐ or five‐membered ring from an imine and a cyclopropane. G: Gibbs‐free energy in kcal/mol. Structure optimization and frequency:M062X/6‐31g*. Single‐point energy with consideration of solvent effect and empirical dispersion correction: M062X/6‐311++G** with SCRF = (SMD, solvent = 1,4‐dioxane) and empirical dispersion = GD3.

We subsequently investigated the origin of the selective seven‐membered ring formation by theoretically comparing the energy profiles of seven‐membered ring formation (Path A, Scheme 2) with those of five‐membered ring formation (Path B). Both reaction pathways commence with the same initial step, namely, the ring opening of the cyclopropane fragment of **RT1(Mg)** via nucleophilic addition of the imine **RT2**. The activation energy barrier for this process is Δ*G* ^‡^ = +18.8 kcal/mol (**TS1**), leading to the formation of the dimethyl malonate enolate derivative **CP1** with an energy gain of –28.9 kcal/mol. Subsequently, along Path A**, CP1** isomerizes to form **CP1‐A** with a slight uphill energy change (Δ*G* = +3.2 kcal/mol). Given that the direct thermal rotation of a C═N double bond is energetically unfavorable, we proposed that the isomerization proceeds through a multi‐step, reversible process involving the interconversion between C═N and C─N bond characters [40]. Specifically, an NTf_2_^–^ anion‐mediated pathway—comprising 1,4‐addition, enamine formation, C─N bond rotation, and subsequent elimination—was considered. Computational analysis of a simplified model reveals that this multi‐step pathway involves two activation barriers, both of which are below 10 kcal/mol (see Supporting Information). This low energy profile indicates that the configurational isomerization occurs readily under the experimental conditions. After the isomerization, the *cis*‐geometry of the two phenyl groups on the C═N bond of **CP1‐A** greatly facilitates access of the C═C bond as the electrophilic site toward the anionic center, thereby enabling intramolecular 1,4‐addition to the azadiene via **TS2‐A** with an activation energy of Δ*G* ^‡^ = +10.7 kcal/mol. The resulting **CP2‐A** (*G* = –10.5 kcal/mol), containing a seven‐membered ring, is energetically more favorable than **CP1‐A** (*G* = –6.9 kcal/mol). Finally, **CP2‐A** releases the azepine product **PD‐A** with a further decrease in free energy (Δ*G* = –2.4 kcal/mol), regenerating **RT1(Mg)** as the active catalytic species.

On the other hand, when the next step from **CP1** proceeds along Path B, a conformational change first takes place to afford **CP1‐B**, in which the iminium C═N bond is orientated toward the anionic center. This step is more energy‐demanding (Δ*G* = +6.2 kcal/mol) than the formation of **CP1‐A**. The following intramolecular nucleophilic addition proceeds via **TS2‐B** with an activation energy computed to be Δ*G* ^‡^ = +19.6 kcal/mol, which is also higher than that for **TS2‐A** (Δ*G* ^‡^ = +10.7 kcal/mol). Alternatively, cyclization of **CP1** could also directly go through a transition state with s‐*trans* geometry with a higher activation barrier (Δ ***G*** ^‡^ = 23.4 kcal/mol, see Supporting Information). Finally, **TS2‐B** leads to **CP2‐B** (*G* = +9.8 kcal/mol) bearing a pyrrolidine ring motif along the IRCs, which subsequently converts into **PD‐B** (*G* = +3.0 kcal/mol), thereby closing the catalytic cycle.

**FIGURE 3:**
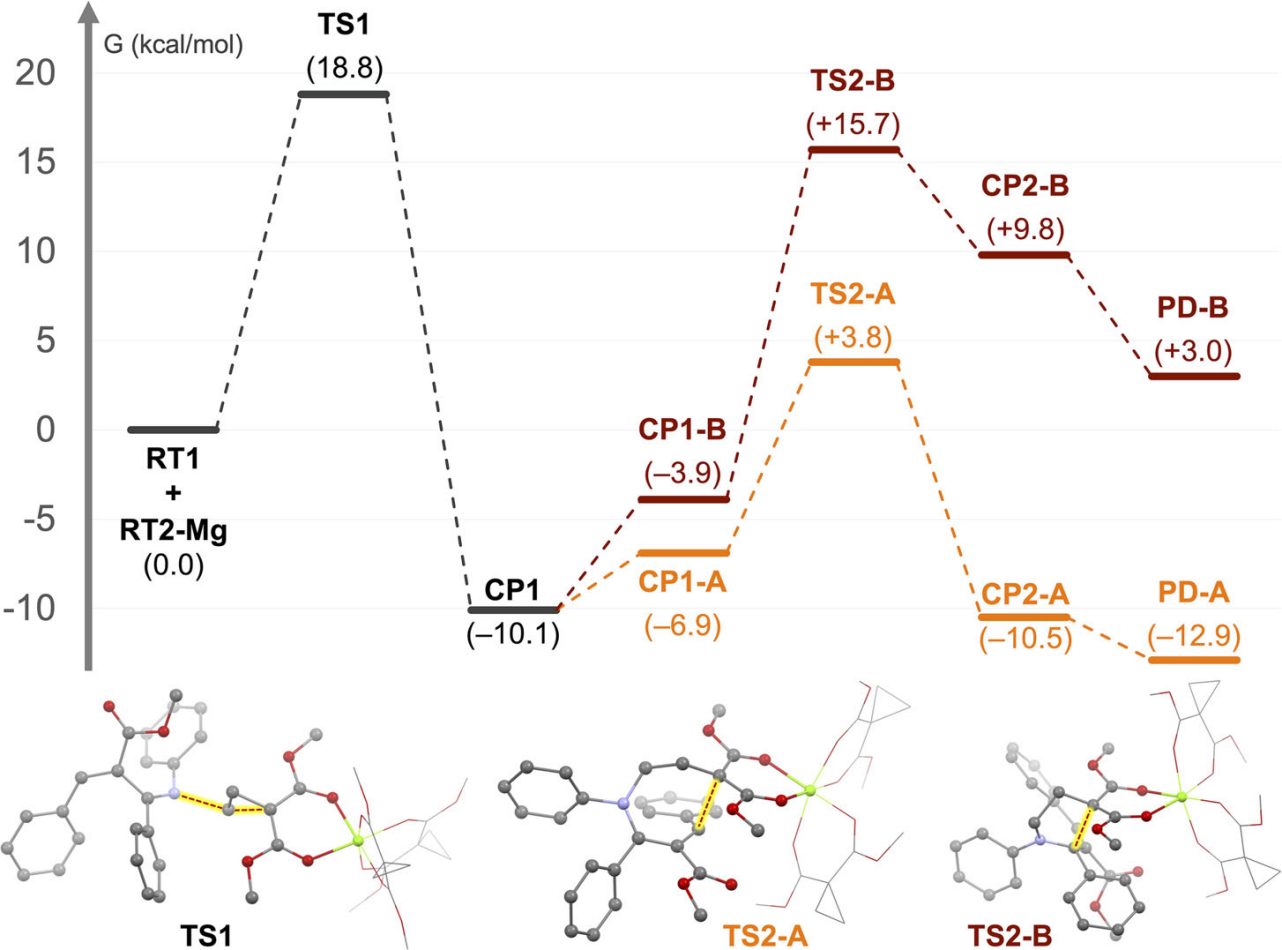
Energy profiles and three‐dimensional representations of all transition states. Hydrogen atoms are omitted for clarity.

The energy diagrams of both reaction pathways are summarized in Figure 3. Notably, relative to Path A, Path B is not only kinetically disfavored due to its higher activation barriers, but also thermodynamically unfavorable as a highly endothermic transformation. In contrast, Path A produces **PD‐A** exothermically with viable kinetic barriers, which is in good agreement with the experimental observation that the generation of azepine (**PD‐A**) occurs under mild conditions and is strongly preferred over the formation of pyrrolidine **(PD‐B)**.

**FIGURE 4:**
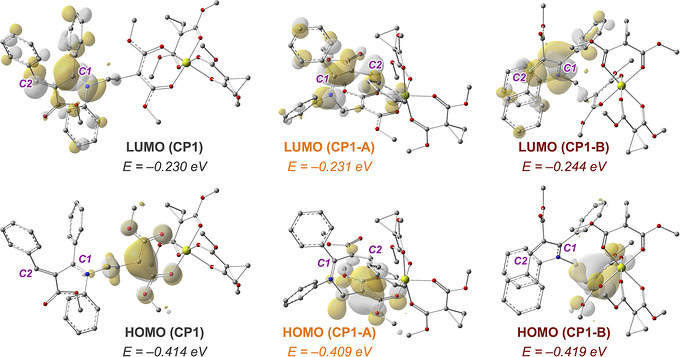
Frontier orbital analysis based on optimized structure (M062X/6‐31g*). Iso‐value = 0.034. Hydrogen atoms are omitted for clarity.

**FIGURE 5:**
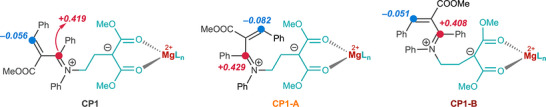
Natural population analysis.

To shed light on the origin of the observed selectivity, frontier molecular orbital analyses were performed for **CP1**, **CP1‐A**, and **CP1‐B**. As anticipated, the HOMOs of all three structures are predominantly localized on the malonate enolate moiety, whereas the corresponding LUMOs are mainly distributed over the azabutadiene fragment. Considering the kinetic and thermodynamic preferences of **Path A** and **Path B**, preferential LUMO localization at C2 (the C═C bond) rather than at C1 (the C═N bond) would be expected, as such polarization should facilitate seven‐membered ring formation. However, the LUMOs of **CP1** and related structures exhibit comparable contributions from both the C═C and C═N bonds, with no pronounced differentiation between C1 and C2. Furthermore, natural population analysis was conducted to evaluate the charge distribution at C1 and C2. Notably, C1 bears more positive charge than C2, which is inconsistent with the experimentally observed selectivity. Taken together, these results indicate that the unusual regioselectivity cannot be rationalized solely on the basis of frontier orbital characteristics or charge distribution, and the factors other than electronic effects, such as steric effects, likely play a dominant role in determining the reaction outcome (Figures 4 and 5).

**FIGURE 6:**
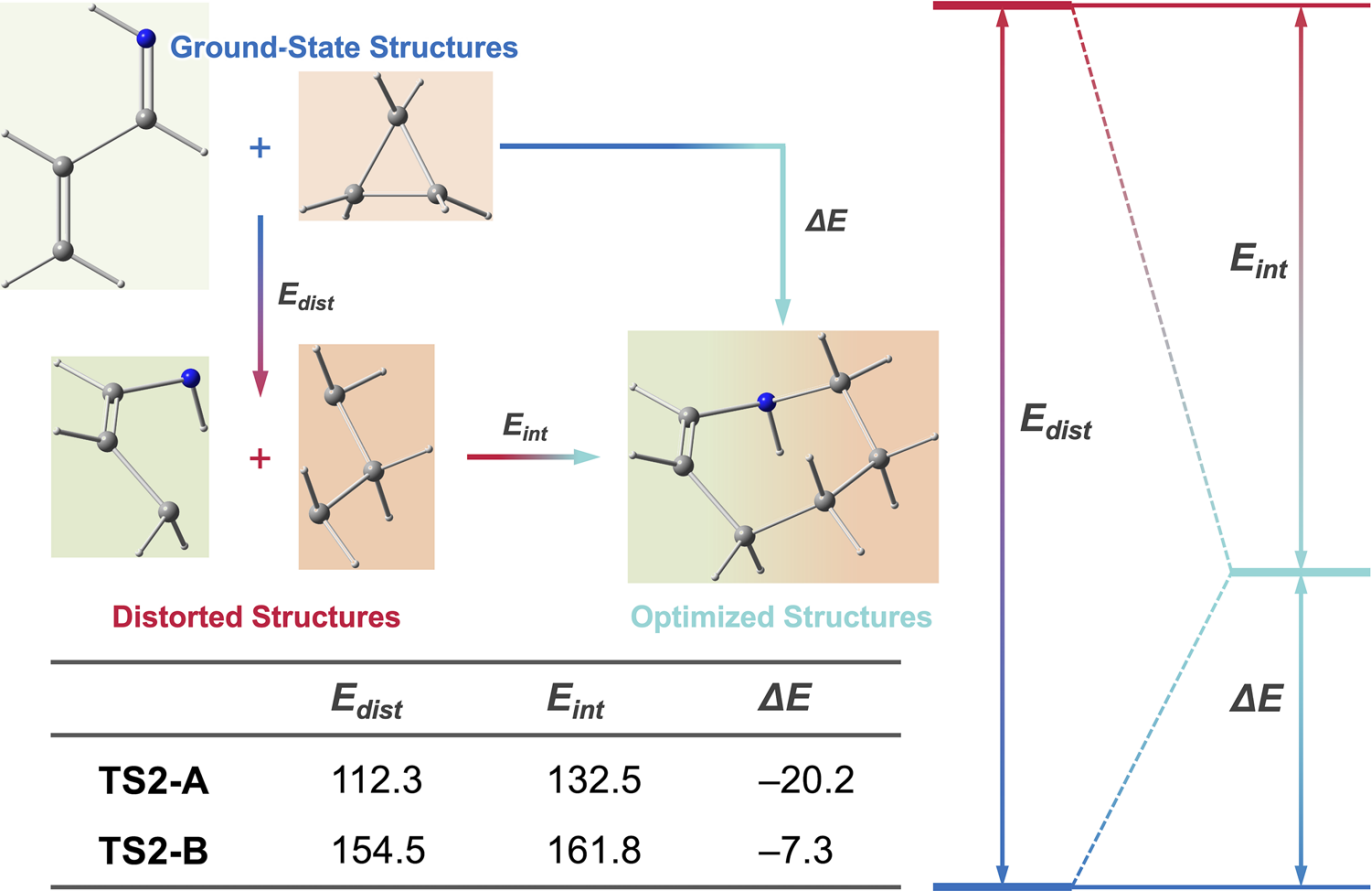
Energy decomposition analysis.

To further examine whether the observed selectivity is governed by steric effects, energy decomposition analyzes allowing quantitative evaluation of structural distortion were performed on **TS2‐A** and **TS2‐B** (Figure 6). As a result, **TS2‐A** exhibits a larger interaction energy (*E*_int_) than **TS2‐B**, which is consistent with the frontier molecular orbital and charge distribution analyses, indicating that interactions arising from orbital and electronic characteristics indeed favor Path B. However, in sharp contrast, the distortion energy (*E*_dist_) of **TS2‐B** is substantially higher than that of **TS2‐A**, thereby exerting a decisive influence on the relative stability of the transition states. This difference can be clearly attributed to increased steric repulsion among the substituents in **TS2‐B**, arising from the more congested molecular environment associated with five‐membered ring formation.

## 3 Conclusion

In summary, a comprehensive DFT study was conducted to elucidate the mechanistic origin of the unusual chemo‐ and regioselectivity observed in the Au/Mg orthogonal relay‐catalyzed synthesis of azepines. Detailed analysis of the reaction pathways revealed that formation of the seven‐membered azepine proceeds via a kinetically and thermodynamically favored pathway, whereas the competing five‐membered ring formation is both kinetically disfavored and highly endothermic. Frontier molecular orbital and natural population analyzes indicate that simple electronic factors, including orbital localization and charge distribution, are insufficient to account for the observed regioselectivity. Instead, energy decomposition analysis of the key cyclization transition states demonstrates that distortion energy is the decisive factor governing selectivity. Specifically, formation of the five‐membered ring requires substantially greater structural distortion due to severe steric congestion in the transition state, whereas the seven‐membered ring pathway proceeds with a significantly lower steric penalty.

The present study not only provides a clear mechanistic rationale for the counterintuitive selectivity observed experimentally but also offers valuable insights into the design of medium‐sized ring formations through relay catalysis, thereby contributing to the rational development of new synthetic strategies for azepine and related heterocycles. These findings further establish that steric effects, rather than electronic preferences, can play a dominant role in directing reaction outcome in Lewis acid‐catalyzed system. Importantly, the present calculations reveal that judicious exploitation of steric effects enables novel reactivity and selectivity—unattainable by reported known methods—without the need for elaborate ligands or specially designed catalysts. Instead, readily available and commercially accessible reagents are sufficient to induce such unconventional reaction outcomes. Overall, these results provide a practical and conceptually new strategy for the design of catalytic reactions and synthetic routes. Ongoing studies are directed toward the development of new heterocycle‐forming reactions and Lewis acid‐catalyzed transformations in combination with computational investigations.

## Conflicts of Interest

The authors declare no conflicts of interest.

## Supporting information




**Supporting File**: chem70960‐sup‐0001‐SuppMat.pdf.

## Data Availability

The data that supports the findings of this study are available in the Supporting Information of this article.
