# Supplementary material for: A DFT Study on the Mechanism of Selective Formation of Substituted Azepines From 1‐Azabutadienes and Cyclopropanes
Source: Chemistry. 2026 Apr 3;32(23):e70960. doi: 10.1002/chem.70960 (PMC13282907; doi:10.1002/chem.70960)
Supplement: Supplementary file 1 — Supporting File: chem70960‐sup‐0001‐SuppMat.pdf. [file CHEM-32-e70960-s001.pdf]

Supporting Information

A DFT Study on the Mechanism of Selective Formation of Substituted Azepines from 1-Azabutadienes and Cyclopropanes

Ryo Kobayashi,[a] Chao Wang,[a] Shuto Kosuge,[b] Yuji Matsuya,[c] Kenji Sugimoto,*[d] and Keiichi Hirano*[a]

[a] Institute of Medical, Pharmaceutical, and Health Sciences, Kanazawa University,
Kakuma-machi, Kanazawa 920–1192, Japan
E-mail: k1hirano@p.kanazawa-u.ac.jp

[b] Graduate School of Pharmaceutical Sciences, Tohoku University
6-3 Aoba, Aramaki, Aoba-ku, Sendai 980-8578, Japan

[c] Graduate School of Medicine and Pharmaceutical Sciences, University of Toyama
Sugitani, Toyama 930-0194, Japan

[d] Department of Biomolecular Chemistry, Faculty of Science and Technology, Kyoto Prefectural University
1-5 Shimogamo Hangi-cho, Sakyo-ku, Kyoto 606-8522, Japan
E-mail: ksugimoto@kpu.ac.jp

Table of Contents

0	Full citation for Gaussian 16	S-2
1	Additional Discussions	S-3
2	Energy Profiles	S-5
3	Cartesian Coordinates	S-6

0. Full citation for Gaussian 16

Gaussian 16, Revision B.01, M. J. Frisch, G. W. Trucks, H. B. Schlegel, G. E. Scuseria, M. A. Robb, J. R. Cheeseman, G. Scalmani, V. Barone, G. A. Petersson, H. Nakatsuji, X. Li, M. Caricato, A. V. Marenich, J. Bloino, B. G. Janesko, R. Gomperts, B. Mennucci, H. P. Hratchian, J. V. Ortiz, A. F. Izmaylov, J. L. Sonnenberg, D. Williams-Young, F. Ding, F. Lipparini, F. Egidi, J. Goings, B. Peng, A. Petrone, T. Henderson, D. Ranasinghe, V. G. Zakrzewski, J. Gao, N. Rega, G. Zheng, W. Liang, M. Hada, M. Ehara, K. Toyota, R. Fukuda, J. Hasegawa, M. Ishida, T. Nakajima, Y. Honda, O. Kitao, H. Nakai, T. Vreven, K. Throssell, J. A. Montgomery, Jr., J. E. Peralta, F. Ogliaro, M. J. Bearpark, J. J. Heyd, E. N. Brothers, K. N. Kudin, V. N. Staroverov, T. A. Keith, R. Kobayashi, J. Normand, K. Raghavachari, A. P. Rendell, J. C. Burant, S. S. Iyengar, J. Tomasi, M. Cossi, J. M. Millam, M. Klene, C. Adamo, R. Cammi, J. W. Ochterski, R. L. Martin, K. Morokuma, O. Farkas, J. B. Foresman, and D. J. Fox, Gaussian, Inc., Wallingford CT, 2016.

1. Additional Discussions

Because the bis(trifluoromethanesulfonyl)imide anion([NTf_2]$^-$) in Mg(NTf_2)$_2$ bind weakly to Mg^{2+} cation, Mg(NTf_2)$_2$ undergoes extensive solvolysis in solution, giving ion pairs consisting of [NTf_2]$^-$ and solvated Mg^{2+} species, particularly in the presence of bidentate ligands.[38] Our calculations on the formation of such ion pairs show that this process is strongly exothermic (Scheme S1), which is consistent with previous reports.[38-39] Therefore, to reduce the computational cost, we focused on the cationic part of the system, i.e., the coordination of Mg^{2+} with dimethyl cyclopropane-1,1-dicarboxylate (**RT1**).

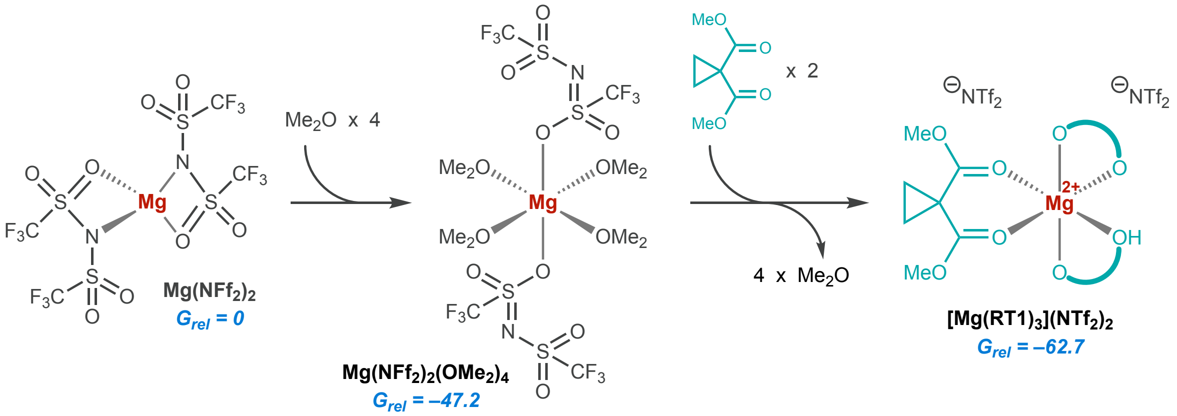

Scheme S1. Calculated energy change for the solvolysis of Mg(NTf_2)$_2$.

The direct thermal rotation of a C=N double in **CP1** bond typically involves a prohibitively high energy barrier. However, in iminium or azabutadiene systems, configurational interconversion is known to proceed under mild conditions through a multi-step, reversible process involving the interconversion between C=N and C–N bond characters,[40] such as addition–elimination pathways or iminium–enamine tautomerization.

To further validate this hypothesis, we performed computational studies using a simplified model with the same calculation method [M062X/6-31g*//M062X/6-31++g**, SCRF = (SMD, solvent = 1,4-dioxane), EmpiricalDispersion = GD3)] (Scheme S2). Specifically, the **RT1(Mg)** moiety in **CP1** or **CP1-A** was represented by a methyl group to maintain the stereochemical environment of the iminium fragment, while $^-$OTf was employed as a computational surrogate for $^-$NTf$_2$, given their similar weak nucleophilicity and good leaving ability. The calculation results reveal that this multi-step pathway (1,4-addition → enamine formation → C–N bond rotation → elimination) involves two activation barriers, both of which are less than 10 kcal/mol. This low energy profile indicates that the configurational isomerization occurs readily under the experimental conditions.

To examine the possible influence of conformational effects, additional searches for alternative transition-state conformations leading to the five-membered ring were performed (Scheme S3). A new transition state **TS2-B'** (G = 13.4 kcal/mol) with *s-trans* geometry was identified with a slightly lower Gibbs free energy than the current *s-cis*-**TS2-B** (G = 15.7 kcal/mol). In this alternative pathway (**Path B'**), cyclization toward the five-membered ring proceeds directly from **CP1** without the initial conformational isomerization of the azabutadiene moiety. However, the actual activation barrier starting from **CP1** to **TS2-B'** ($\Delta G^{\ddagger}$ = 23.4 kcal/mol) is higher than the barrier from **CP1-B** to **TS2-B** ($\Delta G^{\ddagger}$ = 19.6 kcal/mol), and relative Gibbs free energy **CP2-B'** (11.0 kcal/mol) is slightly higher than that of **CP2-B** (9.8 kcal/mol). Crucially, both barriers remain substantially higher than that of the seven-membered ring-forming pathway (**Path A**).

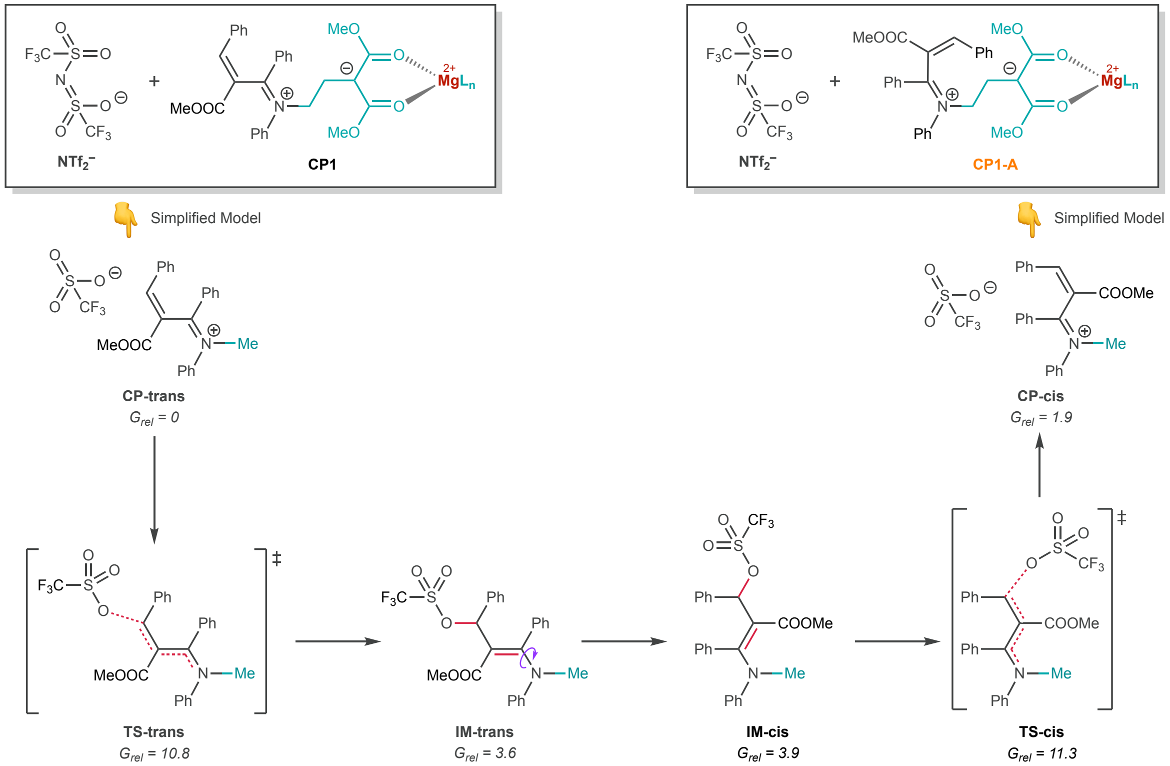

Scheme S2. Calculated pathway for the configurational isomerization of the iminium C=N bond.

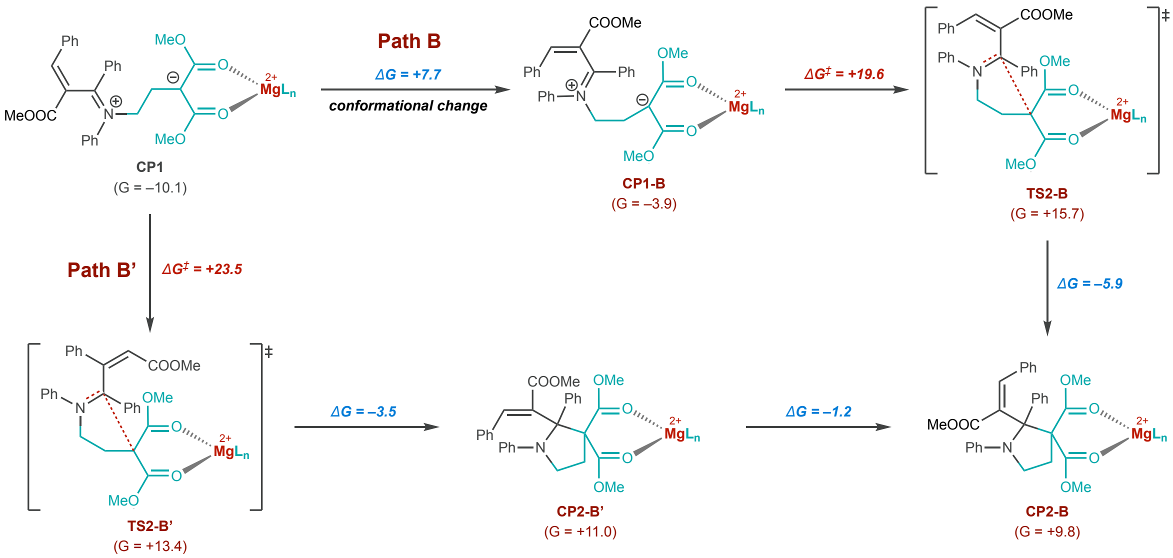

Scheme S3. Conformational analysis of transition states leading to the five-membered ring.

2. Energy Profiles (unit: a. u.)

Gibbs free energy (kcal/mol) used as a basis for discussion was calculated as follows:

G = E-total-SP + G-corr

E-total-SP: *total electronic energy, obtained as single-point energy at M06-2X/6-311++G** with SCRF*

G-corr: *Thermal correction to Gibbs Free Energy, obtained from vibrational frequency at M06-2X/6-31G**

● Starting Materials

ENERGY	Cyclopropane	RT1(Mg-2sol)	RT1(Mg-4sol)	RT1(Mg)	Imine
G-corr	0.133086	0.284613	0.443453	0.446865	0.311424
H-corr	0.183645	0.366087	0.544781	0.560347	0.388528
E-total-SP	-573.594305	-1083.424488	-1393.520032	-1920.7127478	-1092.9291409
G	-573.461219	-1083.139875	-1393.076579	-1920.265883	-1092.617717
H	-573.41066	-1083.058401	-1392.975251	-1920.152401	-1092.540613

ENERGY	Solvent (Me_2O)	$Mg(NTf_2)_2$	$Mg(OMe_2)_4(NTf_2)_2$	$Mg(RT1)_3(NTf_2)_2$	
G-corr	0.056087	0.048721	0.365735	0.530259	
H-corr	0.086593	0.148627	0.506415	0.707629	
E-total-SP	-154.9957438	-3854.423716	-4474.57462	-5575.388816	
G	-154.9396568	-3854.374995	-4474.208885	-5574.858557	
H	-154.9091508	-3854.275089	-4474.068205	-5574.681187	

● CP, TS, and PD

ENERGY	TS1	CP1	CP1-A	TS2-A	CP2-A	PD-A
G-corr	0.781039	0.784929	0.792107	0.796596	0.798919	0.477663
H-corr	0.949558	0.952764	0.953025	0.952237	0.954685	0.577408
E-total-SP	-3013.620365	-3013.669893	-3013.669813	-3013.657057	-3013.682254	-1666.569149
G	-3012.839326	-3012.884964	-3012.877706	-3012.860461	-3012.883334	-1666.091486
H	-3012.670807	-3012.717128	-3012.716787	-3012.704821	-3012.727568	-1665.991741

ENERGY	CP1-B	TS2-B	TS2-B’	CP2-B’	CP2-B	PD-B
G-corr	0.791093	0.794946	0.797100	0.799195	0.795459	0.478346
H-corr	0.952443	0.951612	0.951344	0.952971	0.953224	0.576348
E-total-SP	-3013.663771	-3013.636048	-3013.659314	-3013.665197	-3013.618181	-1666.543958
G	-3012.872677	-3012.841102	-3012.862214	-3012.866003	-3012.822722	-1666.065612
H	-3012.711327	-3012.684436	-3012.707974	-3012.712226	-3012.664957	-1665.967610

● Cis/Trans-Isomerization of C=N Bond in CP1 (simplified model)

ENERGY	CP-trans	TS-trans	IM-trans	IM-cis	TS-cis	CP-cis
G-corr	0.370531	0.371535	0.372491	0.370983	0.371806	0.372802
H-corr	0.470294	0.468861	0.471128	0.470868	0.468961	0.470418
HF-SPE	-2094.251585	-2094.235317	-2094.247783	-2094.245894	-2094.234882	-2094.250902
G	-2093.881054	-2093.863782	-2093.875292	-2093.874911	-2093.863076	-2093.8781
H	-2093.781291	-2093.766456	-2093.776655	-2093.775026	-2093.765921	-2093.780484

3. Cartesian Coordinates

Solvent(Me$_2$O)

O	0.00000000	0.59221500	0.00000200
C	-1.16017300	-0.19538700	-0.00000300
H	-2.01688000	0.48082700	0.00051000
H	-1.21061000	-0.83832500	-0.89207200
H	-1.21013900	-0.83903600	0.89158800
C	1.16017300	-0.19538700	0.00000300
H	1.21063300	-0.83831300	0.89208100
H	1.21011300	-0.83905100	-0.89157800
H	2.01688000	0.48082700	-0.00054300

Imine

C	-1.46387000	-0.01324200	1.81575100
O	-1.82214400	-0.80838600	2.65167200
O	-2.08148000	1.15506300	1.59360500
C	-3.29611800	1.33767400	2.31714500
H	-3.11262500	1.28576700	3.39220400
H	-3.66284900	2.32293200	2.03361900
H	-4.01486900	0.56418200	2.03526500
C	-0.27858900	-0.19307500	0.91747000
C	-0.11110900	0.77141300	-0.21964000
C	0.52952600	-1.22822300	1.20436400
H	0.25237700	-1.80115500	2.08893200
N	-0.97094100	0.90554100	-1.15633800
C	-2.12324200	0.09619800	-1.20680700
C	-2.05919900	-1.29846400	-1.28796100
C	-3.37036000	0.73069900	-1.24053600
C	-3.23084500	-2.04504100	-1.38241200
H	-1.09043900	-1.79010800	-1.28721500
C	-4.53515500	-0.02263800	-1.31250000
H	-3.39973100	1.81478900	-1.19276900
C	-4.47107700	-1.41425200	-1.38571500
H	-3.16983200	-3.12693900	-1.44911900
H	-5.49847200	0.47862900	-1.32314500
H	-5.38185600	-2.00049300	-1.45302100
C	1.09686100	1.64784600	-0.24015900
C	1.96397400	1.72826200	0.85245100
C	1.36452900	2.41527700	-1.38055100
C	3.07806800	2.56193900	0.80733200
H	1.76827200	1.14039600	1.74309800
C	2.47808500	3.24255900	-1.42492100
H	0.68199000	2.34834600	-2.22097800
C	3.33946900	3.31848700	-0.33020900
H	3.74239000	2.61732700	1.66397600
H	2.67751500	3.83070200	-2.31546200
H	4.21073500	3.96537600	-0.36616600
C	1.72472000	-1.69559900	0.49415000
C	2.63637200	-2.48989800	1.20467000
C	1.98929500	-1.41556400	-0.85553400
C	3.79592000	-2.96129400	0.60205800
H	2.42837700	-2.72925500	2.24428300
C	3.14393000	-1.89699000	-1.46024900
H	1.28515500	-0.83474100	-1.44223400
C	4.05339500	-2.66313100	-0.73387000
H	4.49427700	-3.56637200	1.17147100
H	3.33264700	-1.67475800	-2.50565500
H	4.95503100	-3.03448800	-1.21089500

RT1(Cyclopropane)

C	1.26645400	-0.41257300	0.08722400
O	1.35588800	-1.56385500	0.42023100
O	2.34002800	0.35721700	-0.19623800
C	3.59515300	-0.30564700	-0.05367600
H	3.72376700	-0.66175200	0.97073600
H	3.65243700	-1.15860900	-0.73288100
H	4.35217000	0.43655100	-0.30214200
C	-1.26644900	-0.41258500	-0.08717900
O	-1.35587700	-1.56385800	-0.42021800
O	-2.34003100	0.35721100	0.19623600
C	-3.59515200	-0.30565000	0.05363200
H	-3.65246700	-1.15860300	0.73284500
H	-4.35217600	0.43655500	0.30205900
H	-3.72372700	-0.66176700	-0.97078100
C	-0.00000100	0.38096200	0.00001500
C	-0.00211000	1.71288000	0.73992100
C	0.00209800	1.71286500	-0.73992300
H	0.92230000	1.96074600	1.24839300
H	-0.91904500	1.96547900	1.25699100
H	-0.92231300	1.96071400	-1.24840000
H	0.91903100	1.96546000	-1.25699700

Mg(NTf$_2$)$_2$

Mg	0.27758600	0.44576400	-0.95306300
O	-0.93854000	-0.20938400	-2.42550200
O	1.56605800	1.99018800	-0.70710300
S	-2.23554600	-0.19715100	-1.65593200
O	-3.39867300	0.40893000	-2.24584800
C	-2.59633900	-2.01237200	-1.40161300
F	-1.80170200	-2.46508800	-0.44745100
F	-3.85609000	-2.17721000	-1.08339500
F	-2.32682600	-2.61954500	-2.54213900
N	-1.65913300	0.40764900	-0.26613800
S	-2.52039000	0.48545700	1.14518000
O	-3.59914200	-0.47864700	1.10587800
O	-1.55316400	0.55552200	2.21430000
C	-3.27664000	2.16274300	1.00861800
F	-2.31112100	3.06572000	0.93688200
F	-4.02024400	2.21635200	-0.08236300
F	-4.01888300	2.37913600	2.07885900
S	2.67972900	1.21410000	-0.05747800
O	3.19966200	1.64650500	1.21022300
C	4.05057800	1.26590500	-1.29807300
F	3.54338500	1.01044300	-2.49368400
F	4.59149900	2.46688600	-1.28110200
F	4.93832400	0.34808500	-0.97665300
N	2.05962000	-0.27475000	-0.20123600

S	2.57054400	-1.58947000	0.68578200
O	3.84877300	-1.32692800	1.30131500
O	2.29680800	-2.76548400	-0.10294900
C	1.25990600	-1.52786800	1.98498100
F	0.08172400	-1.61889200	1.36227000
F	1.40312800	-2.53692400	2.81843000
F	1.32757100	-0.37812900	2.62829600

$Mg(OMe_2)_4(NTf_2)_2$

Mg	0.00494900	-0.01520600	-0.00488600
O	0.84280500	-0.05141200	-1.93198300
O	0.84901300	-1.80211500	0.74856800
O	-0.81599500	0.00312500	1.93430900
O	-0.83980600	1.75748500	-0.77952800
O	-1.45929900	-1.22215500	-0.75910900
O	1.44912100	1.20535000	0.76268700
C	1.52007600	1.06636900	-2.50126800
H	2.58989900	0.85592700	-2.57292300
H	1.10478900	1.26367400	-3.49687500
H	1.34754700	1.92340400	-1.84997700
C	1.22923500	-1.26195900	-2.57472100
H	0.51356600	-2.02710100	-2.27867500
H	1.17718600	-1.12988300	-3.66072900
H	2.25105400	-1.52642000	-2.28081500
C	0.80134500	-3.10420200	0.17608700
H	0.56968100	-3.83580600	0.95900800
H	0.00151100	-3.11208500	-0.56208500
H	1.76584900	-3.34815000	-0.28095600
C	1.64111600	-1.78087700	1.93572200
H	2.68789400	-1.98364000	1.69606900
H	1.54452200	-0.79385300	2.38485200
H	1.26234800	-2.53810600	2.63239600
C	-1.50313400	-1.11756600	2.48343600
H	-1.34952800	-1.96193800	1.81122200
H	-1.08265800	-1.34428800	3.47063300
H	-2.56932800	-0.89398300	2.56995200
C	-1.16562100	1.19854400	2.62508500
H	-1.07798900	1.03314400	3.70435600
H	-0.45190100	1.96476000	2.32760900
H	-2.19416600	1.48163800	2.37469400
C	-0.88019100	3.03465000	-0.15274600
H	-0.07459300	3.06750500	0.57868000
H	-0.70907800	3.81274000	-0.90549600
H	-1.85489700	3.18813200	0.32103700
C	-1.61739200	1.73492900	-1.97586800
H	-1.48781700	0.75800500	-2.43904100
H	-2.67262800	1.90325100	-1.74556800
H	-1.25267500	2.51547400	-2.65398000
S	-2.94272200	-1.05322000	-0.81478400
O	-3.51653000	-0.90724000	-2.13682500
C	-3.54908600	-2.71075100	-0.18659500
F	-3.76377600	-2.66113200	1.12098600
F	-4.64714200	-3.05766200	-0.81544800
F	-2.59750500	-3.61114500	-0.42741600
N	-3.25919300	0.05155000	0.27630400
S	-4.78641300	0.34702100	0.73692500
O	-5.70808300	-0.70296400	0.33514500
O	-4.74625000	0.83551900	2.10217900
C	-5.22271500	1.81550700	-0.28395700
F	-4.37813800	2.81379700	-0.01994600
F	-5.13385600	1.51517400	-1.57280200
F	-6.45452400	2.20334500	-0.00017300
S	2.93494600	1.06158000	0.82419500
O	3.50406600	0.91672900	2.14842200
C	3.51574100	2.73094700	0.20608300
F	3.73492800	2.68726000	-1.10124300
F	2.55017100	3.61565900	0.44702400
F	4.60718200	3.09250500	0.83844800
N	3.27403800	-0.02877900	-0.27265800
S	4.80636900	-0.30062300	-0.72905100
O	5.71836100	0.74235000	-0.28928300
O	4.78237700	-0.75054200	-2.10799300
C	5.23219900	-1.79874300	0.25187900
F	4.38391300	-2.78422100	-0.04708100
F	6.46393700	-2.18442700	-0.03510800
F	5.13653900	-1.53577400	1.54849600

$Mg(RT1)_3(NTf_2)_2$

C	0.82379900	0.33947400	2.02630000
O	0.64134000	1.30711500	1.28792300
O	1.08974400	0.50748600	3.30599300
C	1.34260500	1.84986600	3.72676900
H	2.17098300	2.25402100	3.14259000
H	0.44633000	2.46034200	3.59646700
H	1.60779100	1.77602400	4.77927100
C	0.52008900	-1.40331800	0.20069500
O	0.22982300	-0.60132000	-0.68324800
O	0.64101800	-2.68270300	-0.03935300
C	0.35781600	-3.12082300	-1.37840800
H	1.02840200	-2.61657400	-2.07336800
H	0.54522800	-4.19247400	-1.36605200
H	-0.68720400	-2.90983900	-1.60585100
Mg	-0.05197900	1.36628400	-0.63145100
O	1.77444300	1.59758400	-1.51136400
O	0.21016100	3.44380900	-0.30913500
C	2.71177900	2.31507900	-1.19062400
C	1.25061700	3.81222900	0.22361400
C	0.73586200	-1.08098900	1.63666300
C	1.51616700	-2.09041900	2.48810900
C	0.05772400	-2.03316900	2.64162900
H	2.14684400	-1.64578300	3.24738300
H	1.96161000	-2.89740800	1.92098700
H	-0.56657600	-2.78378400	2.17203800
H	-0.35725300	-1.54593800	3.51631600
O	-0.95206400	1.48656000	-2.44762000
O	-1.93263700	1.60848600	0.11207800
C	-2.13087900	1.57805500	-2.77499300
C	-2.97870600	1.97099900	-0.41988500
C	-3.25297100	1.93945700	-1.88135700
O	-3.98919700	2.44379900	0.26134100

O	-2.50497300	1.38197500	-4.02391500
C	-3.93371900	2.31819900	1.69091700
H	-3.62355000	1.30566900	1.94741500
H	-3.23304400	3.05546000	2.09003200
H	-4.94969600	2.50524500	2.02871200
C	-1.47751800	0.94616800	-4.92863700
H	-0.69305700	1.70080200	-4.99695300
H	-1.05447100	0.00630200	-4.57065100
H	-1.97730300	0.81251100	-5.88513600
C	-4.66723700	1.50800100	-2.29199900
H	-5.31028800	1.24893800	-1.45670400
H	-4.70925900	0.87036000	-3.16603800
C	-4.29077200	2.91850100	-2.45516500
H	-4.06302700	3.28930500	-3.44790900
H	-4.67647500	3.63927900	-1.74440400
C	2.61804700	3.42804700	-0.20296500
O	3.91202700	2.19531100	-1.70361300
O	1.24390100	4.66709000	1.23353000
C	4.12966500	1.15485600	-2.68340400
H	3.39013000	0.36540000	-2.56560200
H	4.05596800	1.61142700	-3.67211600
H	5.13223600	0.77491400	-2.50052200
C	-0.05462700	5.06364900	1.69867500
H	-0.56157000	5.65291400	0.93273200
H	-0.64697400	4.17585500	1.92751100
H	0.12746200	5.66100300	2.58941000
C	3.62472500	4.58703700	-0.32017200
H	3.22465200	5.56621800	-0.08361500
H	4.28708100	4.52843500	-1.17519700
C	3.78509000	3.60780900	0.76484900
H	4.55563700	2.85289400	0.67869900
H	3.50847400	3.89157800	1.77288800
N	-4.30555600	-0.55478500	0.41805300
S	-3.51008700	-1.91120600	0.09013400
S	-5.91155400	-0.55716900	0.62271700
C	-3.44236600	-1.87848800	-1.75596400
C	-6.08933700	-0.93802300	2.41776100
O	-2.11665900	-1.73002500	0.48396700
O	-4.18145200	-3.15459400	0.41227000
O	-6.35238000	0.82960000	0.51545500
O	-6.63718500	-1.59794900	-0.07977100
N	3.53432600	-0.87362100	-0.12126000
S	4.21974300	-0.40063600	1.24635200
S	4.21524900	-1.89306400	-1.16412400
C	5.90505900	0.29838400	0.89334000
C	4.30485200	-3.56097000	-0.37359200
F	6.20058600	1.13276300	1.89106200
F	5.90386000	1.00205600	-0.23840800
F	6.82034900	-0.64569100	0.83680100
F	3.13001600	-3.88462800	0.16361000
F	4.59872300	-4.44713800	-1.31699600
F	5.23982700	-3.58971900	0.55896000
O	3.46325100	0.77434000	1.69029300
O	4.48569700	-1.46550600	2.20228600
O	3.26744400	-2.06945300	-2.25586300
O	5.60463100	-1.56611600	-1.46038800
F	-3.03176800	-3.05442800	-2.21829600
F	-2.56756000	-0.94706900	-2.17185700
F	-4.62838900	-1.59629200	-2.27786300
F	-5.54303100	0.04253000	3.14086300
F	-5.48394100	-2.07552000	2.72047800
F	-7.37649300	-1.02553700	2.72346700

RT1(Mg-2sol)

C	1.46809200	-1.24417700	-0.28922400
O	0.22407600	-1.35474000	-0.27641600
O	2.22975600	-2.25444800	-0.55855900
C	1.62154600	-3.53957500	-0.86085900
H	1.05142900	-3.87856200	0.00373700
H	0.97994100	-3.43620700	-1.73540200
H	2.45914500	-4.20125800	-1.06238500
C	1.46767400	1.24441000	0.28907700
O	0.22363000	1.35462400	0.27597100
O	2.22899200	2.25488500	0.55863000
C	1.62035100	3.53984300	0.86077800
H	0.97861400	3.43631100	1.73520500
H	2.45771800	4.20177600	1.06244400
H	1.05029200	3.87863700	-0.00393200
Mg	-1.15434100	-0.00028800	-0.00024400
O	-2.39135300	-0.63125700	1.39181600
O	-2.39185600	0.63076100	-1.39181800
C	-2.16903100	-1.78964100	2.22774600
H	-1.26899200	-2.28593400	1.86420900
H	-2.03632400	-1.46679700	3.26259500
H	-3.02737200	-2.45971700	2.14341400
C	-3.58082800	0.09814600	1.76739600
H	-3.67729800	0.94483400	1.08528900
H	-4.44855900	-0.55947800	1.67596000
H	-3.47819800	0.45310300	2.79520700
C	-3.58243000	-0.09758200	-1.76598700
H	-3.67920400	-0.94380900	-1.08335900
H	-4.44937300	0.56102300	-1.67415700
H	-3.48105500	-0.45319000	-2.79369800
C	-2.16892300	1.78830400	-2.22875100
H	-1.26816200	2.28398100	-1.86616300
H	-2.03720900	1.46456200	-3.26344600
H	-3.02653800	2.45930000	-2.14435500
C	2.21840400	0.00021800	0.00003000
C	3.59337600	-0.16977700	0.70918700
C	3.59351900	0.17058900	-0.70875900
H	3.81942500	-1.18870200	1.00211200
H	3.81872200	0.60403100	1.43406100
H	3.81936500	1.18957600	-1.00162300
H	3.81926800	-0.60315600	-1.43357400

RT1(Mg-4sol)

C	-1.87130800	-1.27218000	-0.07459800
O	-0.64115400	-1.36788100	-0.13360300
O	-2.64304300	-2.32573200	-0.04324000
C	-2.01939600	-3.62890900	-0.08130200

H	-1.46313900	-3.73972400	-1.01265700
H	-1.35530600	-3.74069000	0.77656600
H	-2.84160700	-4.33768100	-0.03234000
C	-1.87141300	1.27229700	-0.07458700
O	-0.64125600	1.36804300	-0.13349700
O	-2.64320300	2.32580700	-0.04335600
C	-2.01961600	3.62902200	-0.08149400
H	-1.46311200	3.73969900	-1.01271500
H	-2.84188700	4.33774700	-0.03287900
H	-1.35576200	3.74098500	0.77653200
Mg	0.88106000	0.00024200	0.00048500
O	1.15565900	0.00036900	-2.04428100
O	0.79139700	-0.00010200	2.06152700
C	0.11586700	-0.00011500	-3.02348600
H	-0.83610100	0.00063000	-2.49219500
H	0.19219900	0.89417800	-3.64986000
H	0.19165500	-0.89546500	-3.64842200
C	2.45855300	0.00011400	-2.63666200
H	3.18612200	0.00021600	-1.82319200
H	2.58597200	-0.89410700	-3.25474900
H	2.58612500	0.89404700	-3.25512900
C	0.49573000	-1.18916700	2.80149300
H	0.73112500	-2.03718200	2.15752900
H	1.10540600	-1.22127400	3.70931300
H	-0.56563000	-1.20724200	3.07179600
C	0.49582700	1.18878000	2.80182600
H	0.73129100	2.03697100	2.15812100
H	-0.56553500	1.20687200	3.07211300
H	1.10550000	1.22058200	3.70965700
C	-2.63380500	0.00003300	-0.03883900
C	-4.02714500	-0.00001300	-0.71898200
C	-3.97115900	-0.00000700	0.74208600
H	-4.27946300	-0.92217300	-1.22931900
H	-4.27952900	0.92212300	-1.22933000
H	-4.18532900	0.92219000	1.26955000
H	-4.18527500	-0.92221000	1.26956200
O	2.19778200	1.59360300	0.11132000
O	2.19760100	-1.59355700	0.11081300
C	2.09723200	2.68119200	-0.81667200
H	1.28594000	2.45093700	-1.50641600
H	1.87497000	3.60464700	-0.27327400
H	3.04003600	2.79215900	-1.36150200
C	3.27658300	1.78694800	1.03269300
H	4.23494300	1.65582500	0.52096400
H	3.22237100	2.79333000	1.45897900
H	3.16728100	1.05164500	1.82872000
C	2.09642800	-2.68107100	-0.81718100
H	1.28536900	-2.45026300	-1.50702400
H	3.03921100	-2.79267700	-1.36191300
H	1.87347800	-3.60438400	-0.27381700
C	3.27629400	-1.78756200	1.03218600
H	3.22174900	-2.79407700	1.45812200
H	4.23472000	-1.65658100	0.52054800
H	3.16720100	-1.05251900	1.82848000

RT1(Mg)

C	0.68257600	-2.73391700	-1.07725100
O	0.03492100	-1.69005600	-1.14591600
O	0.57640600	-3.67285500	-1.98572200
C	-0.32944600	-3.44167900	-3.08405100
H	-1.34530400	-3.33270600	-2.70302900
H	-0.02996100	-2.54246100	-3.62287900
H	-0.24247200	-4.32303400	-3.71390200
C	1.87596200	-2.09897200	1.07877400
O	1.37078200	-0.97927600	1.14851300
O	2.71417500	-2.53498900	1.98744500
C	3.02585000	-1.65503000	3.08686900
H	2.11158000	-1.40239800	3.62446000
H	3.70785600	-2.21921400	3.71744500
H	3.50268200	-0.75077000	2.70742200
Mg	-0.00347000	-0.00100300	-0.00020900
O	-1.54086800	-0.69909700	1.14698700
O	-1.48336700	0.81049800	-1.14888400
C	-2.76274900	-0.57587400	1.07563100
C	-2.71121800	0.78079200	-1.07689500
C	1.63876300	-3.09184700	0.00074600
C	1.72732300	-4.58503400	0.38608400
C	2.82733000	-4.00012800	-0.38383400
H	1.02073600	-5.22736600	-0.12589900
H	1.87554100	-4.77241600	1.44284800
H	3.75477100	-3.77313100	0.12828200
H	2.89999000	-4.22820200	-1.44047700
O	0.15975700	1.68389400	1.14407400
O	1.44027400	0.87525900	-1.14882700
C	0.88421300	2.67589600	1.07649100
C	2.02853000	1.95344200	-1.07856100
C	1.86657300	2.96077800	0.00007800
O	2.89830900	2.32509300	-1.98623200
O	0.84815000	3.61957300	1.98563600
C	3.14487900	1.42362500	-3.08467000
H	3.55259400	0.48666100	-2.70396100
H	2.21503100	1.23940300	-3.62324800
H	3.86787200	1.93494800	-3.71467800
C	-0.07394100	3.45646600	3.08256500
H	-1.09474300	3.42579700	2.70028200
H	0.15552200	2.53629200	3.62036500
H	0.07903500	4.32773300	3.71392800
C	3.12072400	3.77479700	0.38759900
H	4.02899700	3.47804000	-0.12327600
H	3.20891600	3.99544100	1.44466600
C	2.06998600	4.44301100	-0.38332400
H	1.41393300	5.13712200	0.12830100
H	2.23388200	4.61937800	-1.43963800
C	-3.50166500	0.13124700	-0.00063600
O	-3.56203100	-1.08657400	1.98087900
O	-3.46908900	1.35159800	-1.98136600
C	-2.95940300	-1.80204600	3.07854600
H	-2.41736200	-2.66765700	2.69657000
H	-2.28171400	-1.14079900	3.61921200
H	-3.79085700	-2.10986100	3.70709600
C	-2.81377800	2.01895100	-3.07899700

H	-2.20440200	2.83865900	-2.69711300
H	-2.19131100	1.30690500	-3.62149600
H	-3.61932400	2.39220300	-3.70584600
C	-4.83748500	0.80320800	0.38625000
H	-5.03788400	1.73884400	-0.12207500
H	-5.07469200	0.76488700	1.44272500
C	-4.88393000	-0.43858100	-0.38831400
H	-5.15431900	-1.35644100	0.12009900
H	-5.11688500	-0.38299300	-1.44496300

TS1

C	-5.35802300	0.78446100	2.26213200
O	-6.23670600	1.22254300	2.95748400
O	-4.06092800	0.75393500	2.63916800
C	-3.80464500	1.40494400	3.88675800
H	-4.38193000	0.93887700	4.68672000
H	-2.73585100	1.29179400	4.06858600
H	-4.07448400	2.46144100	3.81683100
C	-5.56164100	0.16133300	0.91778800
C	-4.35520300	-0.03385400	0.06812800
C	-6.81724200	-0.22225000	0.61167700
H	-7.55426200	-0.05473800	1.39675800
N	-3.57383100	0.93344300	-0.26387100
C	1.02484700	1.20025000	0.81048900
O	2.23475600	1.33495300	0.55755100
O	0.35017200	2.19674000	1.37646900
C	1.07290700	3.40453900	1.65043800
H	1.89262900	3.20318500	2.34137900
H	1.46506900	3.82588600	0.72354400
H	0.34611200	4.07808800	2.09948000
C	0.78413100	-1.09973000	-0.12525100
O	1.97018500	-1.23618600	-0.47464500
O	-0.09713700	-2.06150700	-0.36335200
C	0.37848200	-3.23583000	-1.03292000
H	1.16276600	-3.71246300	-0.44232900
H	-0.49130900	-3.88314500	-1.11778000
H	0.76784700	-2.97459200	-2.01841600
Mg	3.55343400	-0.04104100	-0.10109000
C	0.20636800	0.04929400	0.52766900
C	-1.21516600	-0.02074700	1.06781900
C	-1.45018800	0.55669900	-0.24252800
H	-1.39369000	0.62397400	1.92370200
H	-1.57923100	-1.03583500	1.20583300
H	-1.52519300	-0.10208200	-1.09660500
H	-1.31849400	1.62007400	-0.40186700
C	-3.93244400	2.28111700	0.01331200
C	-5.05774700	2.84448400	-0.59519600
C	-3.13376800	3.06297300	0.85496500
C	-5.38301200	4.17558000	-0.35820900
H	-5.67319200	2.23576600	-1.25126500
C	-3.47425200	4.39111700	1.09341800
H	-2.27470400	2.62205600	1.35251300
C	-4.59490500	4.95296200	0.48674500
H	-6.25981400	4.60356400	-0.83264400
H	-2.86385700	4.98906200	1.76342800
H	-4.85577900	5.98871000	0.67534200
C	-3.99401500	-1.41654400	-0.34038100
C	-4.21013000	-2.48487800	0.53521700
C	-3.44953700	-1.66014900	-1.60665900
C	-3.86423900	-3.77858500	0.15639300
H	-4.64957100	-2.29797200	1.51068600
C	-3.12568500	-2.95558600	-1.99195900
H	-3.33374900	-0.83059100	-2.30026800
C	-3.33065400	-4.01628800	-1.10855900
H	-4.03411400	-4.60411500	0.83969400
H	-2.74149300	-3.14408300	-2.98995800
H	-3.09822500	-5.03163700	-1.41604600
C	-7.32359700	-0.81822500	-0.62267600
C	-8.50174900	-1.57681100	-0.54868400
C	-6.71148700	-0.64229000	-1.87325600
C	-9.02777600	-2.18295900	-1.68206000
H	-8.99878900	-1.69328200	0.41046200
C	-7.24568500	-1.23722100	-3.00845500
H	-5.83367700	-0.00970600	-1.96657700
C	-8.39832800	-2.01595000	-2.91390400
H	-9.93435900	-2.77409500	-1.60820200
H	-6.77230800	-1.08312000	-3.97271800
H	-8.81574800	-2.47692900	-3.80323800
C	5.30020100	-2.51312100	-0.07138800
C	4.32421000	-1.86899600	2.18836500
O	4.85561600	-1.53077800	-0.65754500
O	3.78246400	-0.84783500	1.77719900
C	5.60127500	2.16611300	-0.40413300
O	5.11193700	1.25235600	0.25327500
C	4.25330600	1.65931800	-2.50093200
O	3.62178300	0.73687600	-1.99481800
C	5.11619300	-2.82300100	1.37001700
C	6.26875700	-3.56436300	2.07732900
C	5.04643400	-4.31159800	1.76609800
H	7.14661500	-3.72918800	1.46433500
H	6.44316200	-3.25985600	3.10220500
H	5.06092500	-5.00437800	0.93324000
H	4.35738200	-4.53550700	2.57163300
O	6.01954800	-3.42154900	-0.69051700
O	4.24964400	-2.23156500	3.44944600
C	6.26256000	-3.22518400	-2.09728700
H	6.82985000	-2.30576600	-2.24586200
H	6.83851700	-4.09213600	-2.41026100
H	5.31275200	-3.17035200	-2.62996400
C	3.50088200	-1.37703600	4.33567500
H	3.56035400	-1.85526000	5.30987300
H	3.95285200	-0.38495400	4.35747800
H	2.46736300	-1.30951300	3.99391100
C	5.27370000	2.49358100	-1.81563000
O	6.50927600	2.97141600	0.09891700
C	6.90674500	2.74656200	1.46555300
H	7.63160400	3.52663000	1.68316500
H	6.03697800	2.82459400	2.11850300
H	7.35767700	1.75799800	1.55807200
O	4.07481200	2.01379100	-3.75346500
C	3.10082400	1.27344600	-4.51486600

H	2.11793000	1.38019200	-4.05485100
H	3.11852600	1.71707400	-5.50693900
H	3.38472900	0.22130100	-4.54985000
C	6.41535100	3.06198400	-2.68256100
C	5.36000500	3.97530000	-2.23391300
H	5.57860300	4.66337900	-1.42615000
H	4.60883700	4.28954800	-2.94865500
H	7.38007700	3.10488100	-2.19156600
H	6.41054000	2.73093200	-3.71398400

CP1

C	5.18065200	1.14843800	-2.06602500
O	6.08459500	1.64411100	-2.68364600
O	3.90142500	1.13815100	-2.47822900
C	3.65960300	1.88546700	-3.67796400
H	4.24616700	1.47694500	-4.50196100
H	2.59301700	1.78808100	-3.87470300
H	3.93218400	2.93172700	-3.52268100
C	5.35319900	0.40333600	-0.77250700
C	4.14357800	0.12323300	0.01987800
C	6.59808500	-0.05016000	-0.49747700
H	7.33553100	0.14856100	-1.27488600
N	3.31118500	1.09505900	0.31322800
C	-1.17943300	1.38553700	-0.38819700
O	-2.40829700	1.48390300	-0.15644300
O	-0.50930200	2.49563700	-0.76058600
C	-1.27803600	3.69267200	-0.88784100
H	-2.06335500	3.56669300	-1.63541600
H	-1.72818500	3.96323900	0.06894300
H	-0.57248200	4.46005100	-1.20363000
C	-0.87946600	-0.94077100	0.29346300
O	-2.07523000	-1.19413400	0.58958700
O	0.05491200	-1.87676900	0.54873700
C	-0.40760600	-3.09921000	1.11686900
H	-1.14851000	-3.57052600	0.46602200
H	0.47610200	-3.72924900	1.20543000
H	-0.85543700	-2.92455600	2.09735500
Mg	-3.65137100	-0.05951600	0.06016100
C	-0.35997200	0.23632400	-0.29107600
C	1.10941500	0.33840600	-0.59879900
C	1.87462800	0.84738900	0.63173700
H	1.28068900	1.01279900	-1.44168200
H	1.50656700	-0.64026100	-0.88056900
H	1.79217200	0.12306800	1.43892800
H	1.46940000	1.79923900	0.97427200
C	3.69075100	2.48091400	0.13887800
C	4.87757500	2.93980300	0.70752900
C	2.85260500	3.34175100	-0.57129000
C	5.24288300	4.27136200	0.54006500
H	5.50890300	2.26739600	1.27955500
C	3.23321400	4.66936200	-0.73331600
H	1.91994500	2.98183200	-0.99511700
C	4.42685200	5.13543900	-0.18464400
H	6.16517400	4.63114800	0.98262800
H	2.59379100	5.34315400	-1.29414400
H	4.71669500	6.17233700	-0.31605500
C	3.84139700	-1.28442800	0.35994900
C	4.01331700	-2.25454100	-0.63441900
C	3.46020100	-1.67066500	1.65158600
C	3.77399400	-3.59378400	-0.34739400
H	4.32378300	-1.95254500	-1.63014100
C	3.25531300	-3.01221000	1.94114100
H	3.38207200	-0.92907500	2.44216400
C	3.40479900	-3.97388000	0.94054000
H	3.90020100	-4.34158600	-1.12288200
H	2.99819700	-3.31177700	2.95189400
H	3.25179800	-5.02334400	1.17236600
C	7.09165500	-0.71814600	0.69776400
C	8.22953700	-1.53246900	0.57662800
C	6.51180700	-0.54680600	1.96495500
C	8.73786400	-2.20287500	1.68066300
H	8.70457000	-1.64255700	-0.39422500
C	7.03132000	-1.20300200	3.07166100
H	5.67572100	0.13584700	2.09344400
C	8.13784300	-2.04030000	2.92858800
H	9.60994300	-2.83881000	1.57370300
H	6.58884000	-1.05002500	4.05049300
H	8.54656100	-2.54864000	3.79582200
C	-4.65703800	-2.82207300	-0.46524000
C	-3.11499600	-1.83411500	-2.23221300
O	-4.81871300	-1.76589600	0.13463800
O	-3.31261000	-0.67420900	-1.89355500
C	-6.00139800	1.84060100	0.05014400
O	-5.26454600	1.06069100	-0.54175200
C	-5.08984200	1.27438400	2.35590800
O	-4.27317000	0.45829400	1.94442600
C	-3.76650200	-3.03171700	-1.63876700
C	-4.14734300	-4.15404100	-2.62224500
C	-3.01576400	-4.37061100	-1.71168500
H	-5.08655200	-4.64987400	-2.41023000
H	-3.93819300	-3.93717700	-3.66284200
H	-3.16128400	-5.02166000	-0.85750700
H	-2.01029600	-4.30941600	-2.11090500
O	-5.28053000	-3.92512000	-0.10918200
O	-2.29456700	-2.14045300	-3.21597000
C	-6.15326000	-3.84194800	1.03331400
H	-6.96364200	-3.14251400	0.82547000
H	-6.53610900	-4.84951300	1.17396400
H	-5.58768600	-3.51043900	1.90478400
C	-1.61094700	-1.03760800	-3.84180900
H	-0.95239500	-1.49202200	-4.57786700
H	-2.33669200	-0.37954200	-4.32104200
H	-1.04816200	-0.48604400	-3.08558500
C	-6.02789000	2.06671700	1.51891300
O	-6.88247800	2.58032700	-0.58736100
C	-6.93858700	2.43866600	-2.02021000
H	-7.70321800	3.13927800	-2.34566100
H	-5.96820300	2.68555600	-2.45192800
H	-7.21071400	1.41451000	-2.27724300
O	-5.21996400	1.53531400	3.63896400
C	-4.35221500	0.82203600	4.54028600

H	-3.31176200	1.04417700	4.30119200
H	-4.61288600	1.18314300	5.53183900
H	-4.53275800	-0.24980900	4.45308800
C	-7.38630500	2.43439500	2.14519900
C	-6.37360200	3.48663600	2.00502400
H	-6.49426600	4.21991300	1.21673300
H	-5.83540700	3.80855300	2.88841200
H	-8.22191000	2.42504300	1.45590000
H	-7.56326200	2.01361200	3.12754600

CP1-A

C	-1.84490600	1.85285600	0.14878600
O	-0.81640400	2.35299900	0.55293600
O	-2.67469400	2.43357000	-0.71293500
C	-2.35060900	3.77493200	-1.09584500
H	-1.32786200	3.81927200	-1.46829800
H	-3.06285700	4.03933800	-1.87423300
H	-2.45959800	4.43821000	-0.23504500
C	-2.33996000	0.50525300	0.56115400
C	-3.67622200	0.10865100	0.04443700
C	-1.52224300	-0.24478900	1.32471100
C	-0.18562300	-0.53548600	-1.55044100
H	-0.52900900	0.17520700	1.48505200
C	-2.53903200	-1.60938600	-1.31114200
C	-1.55890000	-0.75893600	-2.15449800
H	-2.06443500	-2.06180800	-0.44332300
H	-1.47174300	-1.24713000	-3.13288300
H	-2.01357300	0.21739700	-2.34728600
N	-3.74893000	-0.87144700	-0.81567700
C	0.53598600	-1.60361300	-0.97300700
O	1.62146200	-1.55536200	-0.33561300
O	-0.04482600	-2.80788400	-1.13862200
C	0.68223800	-3.94907900	-0.68642400
H	1.60718200	-4.05797300	-1.25693500
H	0.92003100	-3.86604000	0.37512300
H	0.02696700	-4.80030000	-0.86343000
C	0.48840100	0.68686200	-1.76198000
O	1.65592500	0.99473800	-1.40978400
O	-0.20529200	1.59893300	-2.47559300
C	0.53100100	2.74824800	-2.88641700
H	1.39009100	2.45613300	-3.49429600
H	-0.16111300	3.34462000	-3.47984300
H	0.88278700	3.31110600	-2.01737300
H	-2.94387500	-2.42555900	-1.90602500
Mg	2.81761500	0.01746800	-0.11676800
O	1.82428000	0.97504000	1.46036300
O	4.17048400	-0.74092000	-1.48764600
C	-5.01232000	-1.40611500	-1.28601400
C	-5.27072500	-1.39127100	-2.65392000
C	-5.90742600	-1.95752700	-0.37711400
C	-6.47411500	-1.91520100	-3.11463000
H	-4.54746100	-0.96202800	-3.34246000
C	-7.10473000	-2.48447600	-0.85201600
H	-5.67205800	-1.97131000	0.68246600
C	-7.39054000	-2.45847800	-2.21514400
H	-6.69429100	-1.90006600	-4.17654200
H	-7.81267400	-2.91889600	-0.15456900
H	-8.32620500	-2.86899500	-2.57912200
C	-4.83713700	0.89580000	0.50148800
C	-4.83453900	1.34326300	1.83097400
C	-5.88322700	1.27455400	-0.35479500
C	-5.88983900	2.10829700	2.31419400
H	-4.01236000	1.07797300	2.49008700
C	-6.91441800	2.06732800	0.12591200
H	-5.87865200	0.97151700	-1.39573500
C	-6.92884000	2.47204000	1.46193400
H	-5.89481400	2.42827200	3.35046700
H	-7.71289700	2.37068500	-0.54254100
H	-7.74723600	3.07982900	1.83382400
C	-1.77497300	-1.55163100	1.92047600
C	-0.67219100	-2.38802100	2.15330900
C	-3.06055800	-1.99820800	2.26757400
C	-0.85840400	-3.66171000	2.67861000
H	0.32424700	-2.03284700	1.89503600
C	-3.23982500	-3.26609900	2.80415200
H	-3.91372200	-1.33196800	2.16658000
C	-2.14050600	-4.10296200	3.00091400
H	-0.00391800	-4.30856600	2.85065000
H	-4.23280100	-3.59945600	3.08742100
H	-2.28360200	-5.09176500	3.42417600
O	3.96926900	1.74027800	-0.13402500
O	4.04339000	-0.89311700	1.25410700
C	1.76394600	2.17892500	1.69422300
C	2.40006000	3.25287200	0.88280500
C	3.43376100	2.84390200	-0.09809900
O	1.11180000	2.67547000	2.72288100
O	3.78522500	3.81854200	-0.90844100
C	0.33427000	1.79726000	3.54624700
H	0.29667400	2.26743700	4.52688100
H	-0.66692700	1.72887000	3.11728300
H	0.80025700	0.81305800	3.60320900
C	4.80464600	3.51125600	-1.87756900
H	4.48286300	2.66417100	-2.48537900
H	4.91029900	4.41157500	-2.47765300
H	5.73833800	3.27208600	-1.36717700
C	2.62230500	4.62516700	1.54637600
C	1.52705200	4.47666900	0.57859000
H	3.52004400	5.14274300	1.22932500
H	2.38827900	4.66946200	2.60244000
H	1.65793100	4.89869500	-0.41093800
H	0.50980700	4.39243800	0.94156400
C	5.16847200	-1.44126000	-1.38015000
C	5.08162100	-1.53897600	1.16033800
C	5.76764400	-1.91180200	-0.10344300
O	5.71259100	-1.98857600	2.22377900
O	5.84197800	-1.85863800	-2.43203900
C	5.13607000	-1.68017800	3.50631000
H	5.09343400	-0.59867000	3.63938500
H	4.13308700	-2.10358600	3.57021800
H	5.80069000	-2.13728300	4.23490000
C	5.34721300	-1.45664200	-3.72313500

H	4.32969700	-1.82456200	-3.85874000
H	5.36192400	-0.36887500	-3.80015800
H	6.02635600	-1.90766000	-4.44208300
C	6.50153100	-3.26569100	-0.12507600
C	7.30240100	-2.03825200	-0.05882600
H	6.38922300	-3.85904800	0.77428400
H	6.44563700	-3.79759300	-1.06708800
H	7.75502400	-1.76444400	0.88647000
H	7.81156600	-1.70383600	-0.95464400

TS2-A

C	-2.00618800	2.00800300	0.04706500
O	-0.99769500	2.51309600	0.52332100
O	-2.87291300	2.70459600	-0.68529300
C	-2.73783500	4.12780700	-0.65004300
H	-1.78229500	4.43798400	-1.07758500
H	-3.56499400	4.51218100	-1.24353600
H	-2.80834800	4.48503200	0.37974000
C	-2.34813600	0.57190400	0.20116800
C	-3.63227200	0.08516900	-0.20938600
C	-1.27906900	-0.18719000	0.70508100
C	-0.31606800	-0.82623100	-1.30134700
H	-0.40295100	0.42786100	0.89114500
C	-2.68045400	-1.96321300	-1.29583200
C	-1.56537100	-1.25005000	-2.06165900
H	-2.29994500	-2.57399100	-0.47327400
H	-1.26043700	-1.93521100	-2.86130900
H	-1.99108500	-0.37742800	-2.55962400
N	-3.79292000	-1.10645600	-0.78415700
C	0.56698200	-1.82288700	-0.77216800
O	1.67797200	-1.63131000	-0.23118800
O	0.06549600	-3.05453900	-0.82666300
C	0.83919300	-4.10547400	-0.23681800
H	1.80362500	-4.19213000	-0.74038400
H	0.99021100	-3.90796700	0.82580400
H	0.25182900	-5.00998800	-0.38024100
C	0.25529600	0.45706600	-1.62429700
O	1.39491400	0.87766800	-1.33978300
O	-0.58152100	1.26258200	-2.27628800
C	-0.10813800	2.57931000	-2.56901000
H	0.76947800	2.53075900	-3.21608500
H	-0.93413600	3.07318400	-3.07824300
H	0.14135300	3.09105200	-1.63583800
H	-3.17067900	-2.65320400	-1.98035000
Mg	2.78213400	0.05184200	-0.15781100
O	1.84988600	1.01738600	1.43660400
O	4.02268600	-0.68904000	-1.62395500
C	-5.09399800	-1.74645000	-0.83209000
C	-5.59047000	-2.18511200	-2.05891300
C	-5.82669000	-1.93458000	0.33824200
C	-6.82932800	-2.81399700	-2.11158300
H	-5.02467100	-2.01284300	-2.97041300
C	-7.06460100	-2.56633900	0.27592600
H	-5.45040900	-1.56406100	1.28708300
C	-7.56647500	-3.00860000	-0.94520300
H	-7.22057300	-3.14651300	-3.06703200
H	-7.63681800	-2.71123600	1.18593700
H	-8.53188200	-3.50101500	-0.98918500
C	-4.82431400	0.93508100	0.02405900
C	-4.97668600	1.54716300	1.27183900
C	-5.76081200	1.16852800	-0.98882300
C	-6.07329300	2.36753300	1.51423300
H	-4.24317300	1.36251200	2.05261800
C	-6.83971200	2.00816600	-0.74999500
H	-5.63177600	0.70814000	-1.96348400
C	-7.00193300	2.60144500	0.50270100
H	-6.20251400	2.82474200	2.48953700
H	-7.55798300	2.20062400	-1.53982400
H	-7.85391900	3.24729900	0.68848200
C	-1.27985300	-1.37552600	1.57475800
C	-0.07954700	-1.65104600	2.25758200
C	-2.40624300	-2.15770000	1.88206900
C	0.00104300	-2.69209900	3.17514400
H	0.78763700	-1.02513400	2.07147900
C	-2.32447300	-3.19779800	2.79768700
H	-3.36228700	-1.94976700	1.41936100
C	-1.11896300	-3.47623900	3.44123900
H	0.93531600	-2.88328100	3.69389400
H	-3.20809800	-3.78721100	3.01876200
H	-1.06144800	-4.28665900	4.16061800
O	3.83958400	1.81196000	-0.30712400
O	4.13278300	-0.78711600	1.12711100
C	1.72356000	2.22347400	1.65207800
C	2.35326500	3.31360600	0.86109500
C	3.41068400	2.94571700	-0.11107800
O	1.00693900	2.68863100	2.64588800
O	3.89288300	3.98767100	-0.75261000
C	0.24188400	1.75913800	3.42888600
H	-0.07768600	2.31873800	4.30495900
H	-0.62089700	1.43570000	2.84399600
H	0.85893500	0.90675800	3.71471800
C	4.93697300	3.73852200	-1.71227200
H	4.58234200	3.03496800	-2.46647800
H	5.15784700	4.70778500	-2.15196700
H	5.81274300	3.33278300	-1.20459300
C	2.48245900	4.69484000	1.53022200
C	1.44254700	4.50881500	0.51283100
H	3.37465000	5.24641300	1.26009100
H	2.19264600	4.72425700	2.57285000
H	1.60941800	4.92791500	-0.47317600
H	0.41103100	4.37443700	0.81924000
C	5.02907300	-1.38726400	-1.58712700
C	5.14192900	-1.46447800	0.95553200
C	5.71760000	-1.85740000	-0.35629800
O	5.84156800	-1.92631500	1.96789000
O	5.62596000	-1.80206100	-2.68333400
C	5.37774200	-1.59946500	3.29165500
H	5.37888000	-0.51713900	3.42445400
H	4.37057000	-1.99262000	3.43504200
H	6.08367100	-2.07647600	3.96658600
C	5.04377800	-1.39999000	-3.93834600

H	4.02366900	-1.77871600	-4.00857600
H	5.04298700	-0.31192300	-4.01101200
H	5.67889300	-1.84208400	-4.70158200
C	6.42915800	-3.22262200	-0.42683800
C	7.25039100	-2.00761800	-0.42916800
H	6.37691400	-3.81041200	0.48166000
H	6.29342400	-3.75758400	-1.35902400
H	7.77780200	-1.73632200	0.47736200
H	7.69382700	-1.68430000	-1.36317700

CP2-A

C	-1.91892300	1.95145900	0.11134200
O	-0.83191500	2.35790400	0.53299800
O	-2.77941500	2.79391400	-0.46296500
C	-2.51742900	4.18647400	-0.30643300
H	-1.59965600	4.47206700	-0.82798000
H	-3.37527200	4.69306400	-0.74490700
H	-2.42542400	4.44191400	0.75176000
C	-2.29850800	0.53295000	0.09755300
C	-3.55588900	0.10920100	-0.31652000
C	-1.11056000	-0.36013200	0.38168000
C	-0.49762200	-0.87036800	-1.02118900
H	-0.33777200	0.30982800	0.76035000
C	-2.78245900	-2.07074500	-1.28828000
C	-1.59691200	-1.40160200	-1.96840500
H	-2.47027200	-2.73026100	-0.47184200
H	-1.15045800	-2.12056300	-2.66082100
H	-1.97767600	-0.57540400	-2.56899500
N	-3.81989600	-1.12959900	-0.82584600
C	0.53658700	-1.92182100	-0.66755900
O	1.67521600	-1.69552200	-0.25808900
O	0.05858100	-3.13587600	-0.74216100
C	0.86524700	-4.20197900	-0.20359500
H	1.81310100	-4.25582500	-0.73985700
H	1.03322100	-4.01504700	0.85856900
H	0.27983400	-5.10528100	-0.35382600
C	0.17459600	0.37569300	-1.60426900
O	1.33467800	0.72844000	-1.40356600
O	-0.63930200	1.11565700	-2.31104900
C	-0.17339200	2.42361700	-2.69075400
H	0.67294200	2.33299000	-3.37251600
H	-1.02355800	2.89425800	-3.17908000
H	0.11384400	2.96396800	-1.78552100
H	-3.26924000	-2.70772400	-2.02730800
Mg	2.78862500	0.02803200	-0.17042500
O	1.84215600	0.91024000	1.41250200
O	3.97394200	-0.69436200	-1.67764000
C	-5.14604300	-1.69984400	-0.75816100
C	-5.72883000	-2.20772600	-1.91935300
C	-5.83692500	-1.76098000	0.45158400
C	-6.99427000	-2.78149800	-1.86762000
H	-5.20395500	-2.13084800	-2.86782200
C	-7.10733200	-2.32745700	0.49502000
H	-5.40110300	-1.33736900	1.35145500
C	-7.68632500	-2.84425700	-0.66026500
H	-7.44445400	-3.16864800	-2.77559100
H	-7.64374000	-2.36443400	1.43729800
H	-8.67526300	-3.28814300	-0.62236300
C	-4.73728100	1.01098400	-0.20364000
C	-5.03356800	1.60489900	1.02471500
C	-5.54986000	1.25968900	-1.31123800
C	-6.13585200	2.44351700	1.14515700
H	-4.40000300	1.39505300	1.88317500
C	-6.64172400	2.11120500	-1.19290100
H	-5.31993300	0.79073700	-2.26380000
C	-6.93787400	2.70126600	0.03497300
H	-6.37310700	2.89252300	2.10417200
H	-7.26676400	2.31116200	-2.05697600
H	-7.79819400	3.35611400	0.12767300
C	-1.23863600	-1.41225800	1.49004800
C	-0.09062100	-1.71372000	2.24057600
C	-2.43661300	-2.01963500	1.87514700
C	-0.12840100	-2.62939500	3.28929900
H	0.84077100	-1.19953000	2.02238700
C	-2.47976800	-2.93319200	2.92577600
H	-3.35778900	-1.78337300	1.36181800
C	-1.32473800	-3.25398100	3.63115200
H	0.77555900	-2.83743400	3.85445500
H	-3.42758800	-3.38915800	3.19372800
H	-1.36085800	-3.96400300	4.45071700
O	3.75136600	1.82089700	-0.34973400
O	4.16147700	-0.82378900	1.06287300
C	1.70978700	2.09866900	1.71775500
C	2.34653300	3.24077000	1.01138600
C	3.38156000	2.94327300	-0.00549900
O	0.99361200	2.48372600	2.73896300
O	3.90584400	4.02817100	-0.52840300
C	0.21324200	1.49412800	3.43450800
H	-0.17899500	2.00691200	4.30937700
H	-0.59614600	1.16201000	2.78254700
H	0.84426900	0.65336900	3.72359600
C	4.92740400	3.85386600	-1.52852700
H	4.53349200	3.26652700	-2.35877000
H	5.18751100	4.85960400	-1.84831900
H	5.78900600	3.35075300	-1.08831200
C	2.48350000	4.56667900	1.78369500
C	1.43858500	4.46576900	0.76119700
H	3.37749600	5.13364800	1.55480700
H	2.19826700	4.51277400	2.82679300
H	1.60077500	4.95954000	-0.19049500
H	0.40866600	4.30245900	1.05799600
C	5.03472900	-1.31311800	-1.67697400
C	5.19597500	-1.45616400	0.85945200
C	5.77213400	-1.77333000	-0.47191700
O	5.92074000	-1.92566300	1.84646400
O	5.64293500	-1.64342700	-2.79266400
C	5.46028100	-1.66777600	3.18880300
H	5.42591100	-0.59208500	3.36343700
H	4.46963100	-2.10243200	3.32569200
H	6.19087800	-2.14579600	3.83596400
C	5.01957400	-1.24467500	-4.02978900

H	4.03706100	-1.71030800	-4.11214700
H	4.92321300	-0.15895000	-4.05859900
H	5.68631400	-1.59944700	-4.81131200
C	6.56633300	-3.08999300	-0.59738000
C	7.31133600	-1.82826300	-0.56944000
H	6.56191700	-3.70919100	0.29160600
H	6.44956600	-3.60132800	-1.54534700
H	7.83347900	-1.55440400	0.33952800
H	7.72069000	-1.44713200	-1.49725500

PD-A

C	-0.37152900	2.33357800	0.92269500
O	-0.94643400	2.46901900	1.97863400
O	0.12262800	3.36691300	0.22007700
C	0.07618800	4.62107800	0.89426600
H	0.68272400	4.57969300	1.80353700
H	0.48792100	5.34807800	0.19606100
H	-0.95158400	4.87468100	1.16143300
C	-0.18820300	1.02217400	0.23142000
C	1.01400600	0.67125000	-0.28524900
C	-1.46894300	0.20447300	0.29126700
C	-1.34580000	-1.29806200	-0.10716100
H	-1.72977300	0.21208500	1.35687400
C	0.04625300	-0.62030500	-2.10918500
C	-1.05036100	-1.54478700	-1.59078700
H	-0.36669200	0.36723000	-2.31556300
H	-1.96555900	-1.38490900	-2.16747600
H	-0.76980600	-2.59575900	-1.70398600
C	2.25674000	1.44814000	-0.01415000
C	3.17398100	1.68233800	-1.04075600
C	2.52783100	1.93863800	1.26775400
C	4.32544900	2.42367300	-0.79843600
H	2.97544200	1.27972300	-2.02975400
C	3.67816300	2.68098200	1.50927400
H	1.83457000	1.72213600	2.07806100
C	4.57889500	2.92809400	0.47498100
H	5.02792200	2.60692100	-1.60556500
H	3.87881800	3.05521300	2.50857000
H	5.48045100	3.50271100	0.66359600
N	1.14121400	-0.41638200	-1.16430400
C	2.16145400	-1.38097500	-1.01973800
C	2.87444700	-1.48853400	0.18427300
C	2.47276700	-2.27143000	-2.05544500
C	3.88484100	-2.43021400	0.32848100
H	2.62816100	-0.83355900	1.01238300
C	3.47729400	-3.21980900	-1.89354500
H	1.93805400	-2.23835300	-2.99682400
C	4.19723400	-3.30611400	-0.70732500
H	4.42299500	-2.48428500	1.27059400
H	3.69475800	-3.89790200	-2.71321500
H	4.97999100	-4.04756400	-0.58818600
C	-2.62116600	0.90253200	-0.43746700
C	-2.40672200	1.81459500	-1.47430200
C	-3.94016400	0.65797600	-0.03801700
C	-3.47764600	2.42626600	-2.12332200
H	-1.39220200	2.08400100	-1.75450400
C	-5.01099800	1.26376100	-0.68456400
H	-4.12777200	0.00386200	0.80931400
C	-4.78379900	2.14472600	-1.73928900
H	-3.28479300	3.13220000	-2.92542800
H	-6.02465300	1.05604500	-0.35596600
H	-5.61853700	2.62074600	-2.24419400
C	-2.66629200	-2.01747600	0.22296800
O	-3.40752800	-2.52307600	-0.57603300
O	-2.88949900	-2.02908800	1.54989700
C	-4.08581600	-2.69996700	1.94829800
H	-4.95690500	-2.21977900	1.49535300
H	-4.05400900	-3.74494400	1.63381000
H	-4.12204700	-2.62094800	3.03326600
C	-0.33666400	-2.07666500	0.75826000
O	-0.00360600	-3.20625200	0.49897800
O	0.04362800	-1.42707100	1.86206800
C	0.78580500	-2.21983400	2.79155100
H	1.65260600	-2.66850700	2.30346900
H	1.08823200	-1.53631800	3.58364400
H	0.14641400	-3.01167100	3.18949300
H	0.43979300	-0.99032000	-3.05812900

CP1-B

C	3.24819500	2.53683600	-1.39838600
O	2.73442000	2.91742100	-2.42241200
O	3.88491000	3.32523400	-0.53224100
C	3.82585000	4.73173700	-0.81851900
H	2.78343700	5.05722800	-0.83363500
H	4.37272000	5.21796200	-0.01392300
H	4.28784400	4.93717000	-1.78486800
C	3.29886100	1.09783900	-0.95549900
C	2.93410200	0.86462900	0.45548600
C	3.65368400	0.21527000	-1.92127700
C	0.46166000	-1.06967700	1.56582000
H	3.88600500	0.67680900	-2.88153600
C	2.84026100	-0.50724800	2.47963100
C	1.72509900	-1.55193400	2.24378200
H	3.63252500	-0.94422900	3.08730300
H	2.12766100	-2.39825400	1.68161900
H	1.50478700	-1.92774300	3.25194800
N	3.50270800	-0.03040600	1.23235100
C	-0.15990800	-1.83346800	0.54755900
O	-1.19972900	-1.54701800	-0.09967100
O	0.43111800	-3.01342600	0.27040000
C	-0.15792600	-3.78600600	-0.77476300
H	-1.22432200	-3.93024500	-0.59099800
H	-0.01671000	-3.29432500	-1.74099400
H	0.35779500	-4.74493200	-0.76181400
C	-0.31750500	-0.04079500	2.16555600
O	-1.40518600	0.44059500	1.76912700
O	0.20940900	0.43661200	3.30274700
C	-0.56787000	1.39701200	4.02298900
H	-1.48645200	0.93570600	4.39145200
H	0.05940000	1.71121900	4.85567800

H	-0.81505900	2.24537600	3.38470600
H	2.42943500	0.34666600	3.00986800
O	-3.98373300	1.30936200	0.55611600
O	-1.85008000	1.22685100	-1.16542700
O	-3.64291100	-1.44135800	1.41457500
O	-3.67819000	-0.95591600	-1.28897900
Mg	-2.54720300	-0.12490400	0.24954100
C	-2.32819400	2.24593400	-1.65416600
C	-4.28174000	2.34733600	-0.02272900
O	-1.77050700	2.86650000	-2.66934100
O	-5.31824000	3.07013800	0.33937200
C	-6.09654000	2.58656900	1.45278900
H	-5.46425600	2.51353300	2.33800200
H	-6.88301000	3.32382700	1.59126800
H	-6.51388100	1.60872400	1.21059600
C	-0.54639400	2.32709000	-3.21349400
H	0.29454900	2.60376600	-2.57505500
H	-0.62441300	1.24324300	-3.29942500
H	-0.43986200	2.79305300	-4.19011000
C	-3.55693000	2.93693800	-1.17999200
C	-3.58483800	4.47295000	-1.31362600
C	-4.40391100	3.67521400	-2.23291500
H	-2.66990400	4.91452600	-1.69043500
H	-4.07719500	4.99162900	-0.49995300
H	-4.06570700	3.55510300	-3.25508000
H	-5.47377600	3.63256700	-2.06727500
C	-4.07347300	-2.11273500	-1.38614500
C	-4.08862800	-2.54377200	1.12248300
C	-4.33205200	-3.04429100	-0.25702100
O	-4.34151400	-2.65831500	-2.55401700
O	-4.41532800	-3.42798600	2.04089200
C	-4.13162500	-1.84221000	-3.72034800
H	-4.37055400	-2.48348000	-4.56493500
H	-4.79615500	-0.97790300	-3.68857100
H	-3.09200900	-1.51423300	-3.75715900
C	-4.20845100	-3.04453000	3.41454700
H	-4.82017400	-2.17276300	3.64835600
H	-4.51639500	-3.90665000	4.00050300
H	-3.15474300	-2.81404200	3.57643500
C	-5.49294100	-4.04213100	-0.43896000
C	-4.11711900	-4.54722300	-0.51014000
H	-6.07009200	-4.23634000	0.45676300
H	-6.05051700	-3.93032000	-1.36081100
H	-3.72530900	-5.09982300	0.33584400
H	-3.70759600	-4.79255800	-1.48305400
C	3.81935600	-1.23252000	-1.87765200
C	2.95627300	-2.06750000	-1.15980500
C	4.89008800	-1.79254200	-2.58968300
C	3.17995900	-3.43780700	-1.12903900
H	2.10994800	-1.63540000	-0.63451300
C	5.12578300	-3.16099100	-2.53570700
H	5.54885900	-1.14800700	-3.16506700
C	4.27058100	-3.98459900	-1.80561300
H	2.51594100	-4.07596800	-0.55584100
H	5.96701300	-3.58632900	-3.07255600
H	4.44824400	-5.05486200	-1.77277200
C	4.81062800	-0.56948800	0.94307800
C	5.81778200	0.31695400	0.55908900
C	5.07620100	-1.92499300	1.12214100
C	7.09204500	-0.17402400	0.30515800
H	5.60447400	1.37903000	0.47292100
C	6.35552600	-2.40475700	0.86123600
H	4.29352300	-2.60601500	1.43694400
C	7.36078100	-1.53482800	0.44671600
H	7.88138800	0.51157900	0.01633200
H	6.56388100	-3.46170900	0.98702500
H	8.35883400	-1.91279400	0.25224100
C	1.96947400	1.84902100	1.00996100
C	0.79144700	2.15174900	0.32554900
C	2.38086200	2.64552700	2.08691800
C	0.02297200	3.24013400	0.72714400
H	0.46771500	1.51618700	-0.49476300
C	1.62043100	3.74356400	2.46769500
H	3.32447200	2.43677300	2.58482600
C	0.44289800	4.04619200	1.78311600
H	-0.90623300	3.46733600	0.21249600
H	1.95348200	4.37243000	3.28672200
H	-0.14618100	4.90907300	2.07744500

TS2-B

C	-1.88284000	-1.41892700	-2.01760000
O	-0.97857200	-1.22946500	-2.79805700
O	-2.63784000	-2.52019600	-2.00617400
C	-2.25146000	-3.57102200	-2.89669200
H	-1.34728400	-4.05501600	-2.51543700
H	-3.07872100	-4.27759100	-2.89751100
H	-2.07368200	-3.17671800	-3.89789000
C	-2.37346800	-0.41857700	-0.99164100
C	-2.30857500	-0.83675500	0.44827800
C	-2.86442200	0.69012500	-1.61048100
C	-0.90476300	0.30593600	1.51961500
H	-2.70450100	0.63499100	-2.68775600
C	-3.02736100	-0.25625300	2.64799200
C	-1.88021800	0.77170000	2.58691500
H	-3.89096400	0.10886500	3.20129400
H	-2.27149900	1.75571500	2.32835100
H	-1.39650900	0.84592900	3.56428300
N	-3.38678600	-0.49031000	1.26120200
C	-0.41822600	1.25788700	0.51129400
O	0.54436900	1.07540500	-0.24834200
O	-1.13936700	2.35391500	0.41661300
C	-0.81131200	3.27628900	-0.63204100
H	0.15471500	3.74161900	-0.41905000
H	-0.77194200	2.75578100	-1.58986700
H	-1.61227900	4.01388600	-0.62436800
C	0.14691800	-0.59354900	2.04959300
O	1.30411600	-0.72888000	1.63351700
O	-0.27498900	-1.30074800	3.07631300
C	0.60648900	-2.31117000	3.59204700
H	1.52966600	-1.85420400	3.95046500
H	0.05980700	-2.77685600	4.40854400

H	0.82183900	-3.03603700	2.80440100
H	-2.67247700	-1.17053900	3.13492300
O	4.11641900	-0.58951500	0.78333000
O	2.28782100	-1.28638000	-1.12941100
O	2.70262700	1.85458500	1.45005900
O	3.22770600	1.43235200	-1.21669900
Mg	2.33828600	0.24887000	0.20301700
C	3.06346600	-2.19951000	-1.40256600
C	4.72732600	-1.59195200	0.42141700
O	2.82550100	-3.03570800	-2.38347000
O	5.84245500	-1.96898000	1.00224900
C	6.32706200	-1.16078100	2.09317200
H	5.57658100	-1.12302900	2.88317900
H	7.23145300	-1.65735900	2.43477900
H	6.54544500	-0.15453000	1.73414100
C	1.60983600	-2.82538700	-3.13956700
H	0.74653200	-2.96458300	-2.48976000
H	1.58935300	-1.81238400	-3.54002800
H	1.63856300	-3.57027900	-3.93080800
C	4.33218700	-2.49564900	-0.68935000
C	4.73506600	-3.97914600	-0.57072100
C	5.47240500	-3.12970900	-1.51114900
H	4.01002800	-4.67958000	-0.96798300
H	5.20788800	-4.24884300	0.36594400
H	5.26698600	-3.22928400	-2.57019900
H	6.46752400	-2.79904600	-1.23867600
C	3.41337900	2.64475100	-1.26719100
C	3.02138600	3.01760300	1.22124600
C	3.37173100	3.57737000	-0.11033800
O	3.70045600	3.25519800	-2.39314400
O	3.07726300	3.91996700	2.17472700
C	3.77387400	2.44377800	-3.58339100
H	3.97783800	3.14206200	-4.39085000
H	4.58209100	1.71885600	-3.48140400
H	2.82415800	1.93008100	-3.73560500
C	2.75071000	3.48817500	3.51071300
H	3.43699000	2.69936300	3.81963500
H	2.86562600	4.37265600	4.13176700
H	1.72353700	3.12192500	3.53691500
C	4.37380400	4.75027100	-0.13659100
C	2.95793000	5.03684000	-0.38711700
H	4.79323900	5.01179100	0.82732000
H	5.05672900	4.74458000	-0.97760900
H	2.37960700	5.50387200	0.40158000
H	2.64204600	5.23506300	-1.40456400
C	-3.58870600	1.92132700	-1.28768100
C	-3.94067800	2.38805000	-0.01174700
C	-3.93243700	2.71820100	-2.39666700
C	-4.60049800	3.59839200	0.14631400
H	-3.67480600	1.80594800	0.85584800
C	-4.59620200	3.92809700	-2.24241800
H	-3.67608600	2.37560000	-3.39580900
C	-4.93167700	4.37308800	-0.96595900
H	-4.86572400	3.93911800	1.14234300
H	-4.85515200	4.51877000	-3.11458000
H	-5.45390800	5.31565200	-0.83693800
C	-4.74798600	-0.55016800	0.83324200
C	-5.13435700	-1.48318700	-0.13336600
C	-5.71395900	0.28486200	1.40660200
C	-6.46228400	-1.54988700	-0.54325600
H	-4.40244000	-2.16208700	-0.55821700
C	-7.04161700	0.19173300	1.00703200
H	-5.43931700	1.01894000	2.15787400
C	-7.42216900	-0.71774700	0.02378200
H	-6.74787100	-2.27678600	-1.29664000
H	-7.77955200	0.84316800	1.46328800
H	-8.45869800	-0.78357200	-0.28829500
C	-1.70517200	-2.20737300	0.66419200
C	-0.47205300	-2.54879400	0.09275400
C	-2.42129000	-3.17861400	1.36432800
C	0.02711200	-3.84050000	0.21413100
H	0.09168600	-1.80202400	-0.46117100
C	-1.91411200	-4.47141000	1.49251700
H	-3.39306200	-2.93414800	1.78406900
C	-0.69248900	-4.80880200	0.91759000
H	0.98156500	-4.09570000	-0.24230900
H	-2.48599800	-5.21953300	2.03163400
H	-0.30676400	-5.81880600	1.01063400

TS2B′

C	-2.38701900	-1.72362500	1.55461800
O	-1.90126500	-1.92357700	2.64607100
O	-2.90944000	-2.69500100	0.80808200
C	-3.02142500	-3.98304600	1.41957200
H	-3.75370400	-4.52526300	0.82302400
H	-2.05747700	-4.49589700	1.39476500
H	-3.35905900	-3.88252600	2.45114700
C	-2.48726000	-0.35643100	0.90771500
C	-2.48672700	-0.28353800	-0.60110300
C	-2.55143600	0.61665300	1.85067700
C	-0.90436700	-1.38838800	-1.25650700
H	-2.41437500	0.19851500	2.84664900
C	-3.01490500	-1.65632700	-2.47616600
C	-1.72045300	-2.38475700	-2.06323600
H	-3.77962100	-2.32988100	-2.85907200
H	-1.97085800	-3.23868300	-1.43228000
H	-1.18068000	-2.74194600	-2.94465500
N	-3.48488600	-1.00689200	-1.26643500
C	-0.12108600	-1.87219100	-0.09370700
O	0.59736500	-1.14439800	0.60486700
O	-0.22987700	-3.15826800	0.17436000
C	0.46693500	-3.63322300	1.34071900
H	1.52644300	-3.37605300	1.27172300
H	0.02763500	-3.18336400	2.23324000
H	0.33232500	-4.71322100	1.33430400
C	-0.05938900	-0.49115900	-2.09622600
O	1.00379800	0.05307800	-1.77332900
O	-0.56540500	-0.29460600	-3.29262400
C	0.07150000	0.68816100	-4.12601400
H	1.09996500	0.39099600	-4.33404700
H	-0.51883100	0.71348500	-5.03885900

H	0.04892200	1.65540600	-3.61949400
H	-2.79504800	-0.92056700	-3.25458800
O	3.69138600	0.94067000	-1.03404700
O	1.70357700	1.70976700	0.68572900
O	2.94061800	-1.88245800	-0.84667200
O	3.33995300	-0.52529500	1.50333400
Mg	2.17586800	-0.09787700	-0.12835100
C	2.14347000	2.84798500	0.53771100
C	3.98371500	2.13362000	-1.06844100
O	1.61639800	3.88351300	1.14567300
O	4.97929800	2.57999600	-1.79780600
C	5.71762100	1.61582600	-2.57474200
H	5.04286300	1.10455700	-3.26182600
H	6.46174700	2.19563300	-3.11446500
H	6.19253200	0.89530100	-1.90805700
C	0.45798600	3.66704000	1.98703000
H	-0.42497200	3.53094700	1.35908800
H	0.61383300	2.79125800	2.61699200
H	0.36540400	4.57270900	2.58145900
C	3.29631000	3.21932000	-0.32258000
C	3.25349400	4.60899900	-0.99033200
C	4.16427900	4.40755800	0.14041800
H	2.33905900	5.16577500	-0.82327000
H	3.66781700	4.64516100	-1.99066200
H	3.89144900	4.81929000	1.10467700
H	5.22376200	4.30208600	-0.05994000
C	4.01124800	-1.49881100	1.83502100
C	3.65160100	-2.75233100	-0.35078900
C	4.26937700	-2.70105600	0.99940300
O	4.59206700	-1.57191800	3.00896500
O	3.93245200	-3.86362400	-0.99146300
C	4.39545200	-0.46788800	3.91629800
H	4.92639900	-0.74753300	4.82241400
H	4.81489900	0.44029400	3.48232100
H	3.33011500	-0.33745300	4.10809200
C	3.35452200	-4.03160500	-2.30202600
H	3.69700800	-3.23271500	-2.96009100
H	3.70648200	-5.00057900	-2.64612500
H	2.26613100	-4.01430600	-2.22465900
C	5.63149200	-3.40209200	1.17733600
C	4.44041500	-4.04449700	1.74054400
H	6.05589300	-3.80641000	0.26607700
H	6.31609800	-2.89972700	1.85028900
H	4.02507900	-4.90345000	1.22660700
H	4.28290200	-3.99530400	2.81149000
C	-2.78715400	2.06430400	1.93396800
C	-3.41019800	2.88093200	0.97665700
C	-2.40981600	2.65370900	3.15651700
C	-3.59502600	4.23796400	1.21512100
H	-3.76545600	2.46408200	0.04318500
C	-2.58455400	4.01192900	3.39160000
H	-1.97836600	2.02680900	3.93302700
C	-3.17282100	4.81182500	2.41325400
H	-4.08468500	4.84760900	0.46268700
H	-2.28902300	4.43953000	4.34450900
H	-3.32853100	5.87046900	2.59465100
C	-4.73496700	-1.34959800	-0.68045800
C	-5.44270000	-0.37447500	0.02422000
C	-5.29245300	-2.62275800	-0.83694700
C	-6.67972900	-0.67585900	0.58759300
H	-5.03279100	0.62677500	0.11791800
C	-6.53845100	-2.90873600	-0.29324100
H	-4.74597200	-3.40019400	-1.36286200
C	-7.23565000	-1.94048900	0.42827900
H	-7.21815100	0.09260900	1.13252500
H	-6.96330600	-3.89850100	-0.42659900
H	-8.20675700	-2.16957000	0.85317300
C	-2.17330000	1.05992300	-1.20988900
C	-1.09975800	1.82364100	-0.74598000
C	-2.99278600	1.57688900	-2.21832600
C	-0.85018800	3.08264500	-1.28265000
H	-0.48073800	1.43302200	0.05654900
C	-2.74631800	2.84135500	-2.74839700
H	-3.84976100	1.00237100	-2.55644200
C	-1.67436200	3.59942700	-2.28201200
H	-0.02193600	3.68082600	-0.90888000
H	-3.40053300	3.23811800	-3.51790800
H	-1.48847800	4.58833200	-2.68869200

CP2-B′

C	-2.49748471	-1.92969931	1.49552250
O	-2.06094200	-2.17715216	2.59984097
O	-3.03666797	-2.86620675	0.71575943
C	-3.14246864	-4.16925940	1.29632467
H	-3.72108547	-4.75823970	0.58691464
H	-2.14818453	-4.60211613	1.43571702
H	-3.65069836	-4.11298505	2.25933164
C	-2.48553754	-0.54426379	0.87427503
C	-2.34618738	-0.46487468	-0.65490230
C	-2.59465919	0.40236850	1.83544818
C	-0.99050850	-1.36510087	-1.14643491
H	-2.58408934	-0.05934284	2.82122210
C	-2.94969068	-1.96539814	-2.44710478
C	-1.61204152	-2.52072203	-1.95780367
H	-3.65499613	-2.76180574	-2.67674692
H	-1.79095251	-3.36226979	-1.29139304
H	-0.96213323	-2.84420511	-2.77468619
N	-3.41926904	-1.15440425	-1.34272722
C	-0.13754053	-1.79649405	0.03789077
O	0.57208084	-1.02357162	0.68148446
O	-0.20087759	-3.07137174	0.33282397
C	0.51600264	-3.50524122	1.50678935
H	1.56416283	-3.20864285	1.42913389
H	0.05209629	-3.05669965	2.38688219
H	0.41547875	-4.58842267	1.51631798
C	-0.09990545	-0.52014931	-2.06084054
O	1.00500381	-0.05375292	-1.78525827
O	-0.63886992	-0.34252882	-3.23590662
C	-0.01196268	0.60829978	-4.11761767
H	0.99787768	0.27821585	-4.36258196
H	-0.64240018	0.63301714	-5.00281127

H	0.00816116	1.58237246	-3.62375425
H	-2.81700746	-1.36800012	-3.35798621
O	3.65479573	0.96981563	-1.09276355
O	1.70353214	1.73179239	0.66811904
O	2.91660380	-1.89369754	-0.76315564
O	3.37661068	-0.44343894	1.50853073
Mg	2.19169282	-0.07101878	-0.12141443
C	2.12237217	2.87590252	0.49927687
C	3.91554998	2.17023991	-1.16287148
O	1.60645086	3.90317833	1.12613095
O	4.86967990	2.62322446	-1.93998258
C	5.60269469	1.66192694	-2.72619572
H	4.91553336	1.12059479	-3.37695236
H	6.31047750	2.24929739	-3.30511677
H	6.12097190	0.96742368	-2.06430442
C	0.49146054	3.66421470	2.02239150
H	-0.40393936	3.43711553	1.43958287
H	0.72719382	2.83788324	2.69279998
H	0.36538044	4.59542689	2.56864035
C	3.23280336	3.25489451	-0.41142994
C	3.13176059	4.63134890	-1.10152944
C	4.09154554	4.47109521	-0.00577742
H	2.21145424	5.16955634	-0.90727628
H	3.50405750	4.65827779	-2.11859200
H	3.84858282	4.89385964	0.96172181
H	5.14418538	4.38540429	-0.24753912
C	4.08586094	-1.38661651	1.85138309
C	3.68091756	-2.72323935	-0.27337210
C	4.34696136	-2.60982301	1.04842643
O	4.70649340	-1.40328263	3.00578070
O	3.97585294	-3.84051904	-0.89400544
C	4.51560349	-0.27168239	3.88083569
H	5.08690557	-0.50568018	4.77519584
H	4.89556351	0.62974186	3.39913788
H	3.45517632	-0.16047943	4.10837070
C	3.35787037	-4.06829399	-2.17745100
H	3.64554478	-3.27639546	-2.86928337
H	3.73455396	-5.03290676	-2.50718959
H	2.27325110	-4.09047391	-2.05989751
C	5.73252853	-3.27067322	1.20095827
C	4.57997433	-3.92370251	1.82721489
H	6.13568010	-3.69415729	0.28874072
H	6.42615964	-2.72777692	1.83191873
H	4.16963058	-4.80975881	1.35709876
H	4.45800604	-3.84201265	2.90084125
C	-2.72089348	1.86561262	1.99173513
C	-3.26446589	2.79212809	1.08785631
C	-2.32362718	2.34437228	3.25584682
C	-3.36493699	4.13867054	1.42364788
H	-3.62235355	2.47786857	0.11629475
C	-2.41078567	3.69109559	3.58780014
H	-1.94940758	1.63754033	3.99210902
C	-2.93177084	4.59733813	2.66576799
H	-3.79913844	4.83051895	0.70900667
H	-2.10282958	4.02624179	4.57333973
H	-3.02538043	5.64765029	2.92314774
C	-4.74242981	-1.22223896	-0.84767872
C	-5.32153439	-0.11039489	-0.22784217
C	-5.50570896	-2.39200808	-0.96632023
C	-6.62046552	-0.16853620	0.26988853
H	-4.75797284	0.81059537	-0.13774395
C	-6.81012194	-2.43442350	-0.49062326
H	-5.08197510	-3.28429438	-1.41493430
C	-7.37707217	-1.32633238	0.13566999
H	-7.04339364	0.70865641	0.74916923
H	-7.38387043	-3.34921882	-0.60064825
H	-8.39426242	-1.36674310	0.50921814
C	-2.14968342	0.96181174	-1.17773481
C	-1.10654406	1.75715624	-0.70134217
C	-2.98488497	1.46223880	-2.17840310
C	-0.90298617	3.03542022	-1.21581481
H	-0.47593136	1.38208849	0.10026459
C	-2.79426922	2.74970270	-2.67766892
H	-3.79882046	0.84583888	-2.54708824
C	-1.75130388	3.54077748	-2.19947979
H	-0.09924466	3.65914191	-0.83034245
H	-3.46359746	3.13431670	-3.44047270
H	-1.60561845	4.54403630	-2.58702971

CP2-B

C	-1.54632700	1.60139000	1.12356900
O	-0.93817100	2.37459800	0.40690800
O	-2.05807100	1.95124300	2.30497200
C	-2.12212200	3.35197300	2.57042600
H	-2.68424000	3.84831000	1.77486700
H	-2.63625200	3.44921100	3.52483800
H	-1.12167700	3.78109200	2.63156400
C	-1.82696100	0.16170300	0.78337700
C	-2.34402500	0.00034800	-0.66005800
C	-1.40921800	-0.70133500	1.73245600
C	-1.02812200	-0.26784600	-1.57408600
H	-0.98627000	-0.18873800	2.59682200
C	-2.96215400	-1.46033000	-2.44169200
C	-1.62777900	-0.83935800	-2.88023900
H	-2.99899500	-2.53483800	-2.63738800
H	-0.96136800	-1.57804700	-3.32586100
H	-1.76822900	-0.04877600	-3.61341700
N	-3.09169200	-1.23681100	-0.99310200
C	-0.17746700	-1.39190200	-0.94436000
O	0.84151800	-1.24314300	-0.27589900
O	-0.60783400	-2.59057300	-1.23336500
C	0.18226900	-3.70097600	-0.76312000
H	1.12846900	-3.71407900	-1.30953400
H	0.35905300	-3.60926200	0.30889700
H	-0.40876600	-4.58509100	-0.98725100
C	-0.17063900	0.97961800	-1.79556700
O	0.98442300	1.15582500	-1.41515300
O	-0.78735100	1.87271000	-2.51534800
C	-0.17821400	3.16679400	-2.63605600
H	0.75674000	3.08783500	-3.19293800
H	-0.90428600	3.77161500	-3.17432200

H	-0.00491700	3.56185100	-1.63403500
H	-3.78130000	-0.97926700	-2.99126400
O	3.48155300	1.80671500	-0.22611400
O	1.54337200	1.07017200	1.48098900
O	3.29712400	-0.64386800	-1.74814200
O	3.45941800	-1.01408600	0.96456200
Mg	2.25820200	0.17205900	-0.17416800
C	1.58644600	2.19473700	1.96129200
C	3.19669300	2.94438000	0.13415900
O	0.86076700	2.36108600	3.05328900
O	3.69778200	3.99712500	-0.47091200
C	4.58385100	3.75792300	-1.58328600
H	4.06487200	3.17400600	-2.34431400
H	4.84651400	4.74508400	-1.95452600
H	5.46822600	3.22244600	-1.23696800
C	1.03226900	3.30508700	4.12064400
H	2.06127300	3.28983200	4.48281800
H	0.72882200	4.31068800	3.82354600
H	0.36721900	2.94735700	4.90478200
C	2.30205300	3.31802400	1.27621400
C	1.48124000	4.60861300	1.07898000
C	2.66756400	4.65758000	1.94220600
H	0.48778800	4.58091400	1.51372200
H	1.54000500	5.04794600	0.09033400
H	2.56871100	4.74292400	3.01213200
H	3.56301100	5.11245500	1.53817600
C	4.26497900	-1.90099500	0.69377700
C	4.20285300	-1.47210200	-1.81111900
C	4.77324500	-2.22293600	-0.66409700
O	4.78501500	-2.66832600	1.62166200
O	4.76440100	-1.79639200	-2.95262600
C	4.35488800	-2.44380900	2.98010200
H	4.89094900	-3.18058600	3.57255600
H	4.61658400	-1.43016100	3.28455800
H	3.27620500	-2.59035800	3.04798800
C	4.28334800	-1.13669300	-4.14032000
H	4.42093100	-0.05949200	-4.04267300
H	4.88619500	-1.53608200	-4.95166900
H	3.22775700	-1.36781600	-4.28699000
C	6.24995900	-2.65857400	-0.76474100
C	5.21015500	-3.67769800	-0.93330300
H	6.77143700	-2.29789600	-1.64315100
H	6.79591100	-2.62563700	0.17060100
H	4.99813200	-4.03818500	-1.93290700
H	5.02150200	-4.36646600	-0.11861400
C	-3.27150100	1.19614900	-1.02725300
C	-4.08572000	1.71299300	-0.00621700
C	-3.54982800	1.63026800	-2.33047300
C	-5.08212300	2.65087300	-0.26130800
H	-3.97583400	1.36478800	1.01359400
C	-4.54482700	2.56944000	-2.58876900
H	-2.98806400	1.26965600	-3.17779400
C	-5.31384000	3.09451200	-1.55690900
H	-5.69017600	3.01457000	0.56094400
H	-4.71974600	2.88282300	-3.61307800
H	-6.09184500	3.82191600	-1.76273700
C	-4.40917200	-1.45023500	-0.47600900
C	-5.41304500	-2.00337300	-1.28292800
C	-4.71630600	-1.20728600	0.87315800
C	-6.67769100	-2.27615700	-0.76415800
H	-5.23286100	-2.24258500	-2.32235900
C	-5.98287000	-1.47202500	1.37693500
H	-3.96202200	-0.82141900	1.54640200
C	-6.97840200	-2.00557500	0.56317400
H	-7.43012000	-2.70387100	-1.41883200
H	-6.18497100	-1.26766200	2.42361800
H	-7.96500500	-2.21543700	0.96118900
C	-1.31039400	-2.16448700	1.91055000
C	-0.30989200	-2.58028900	2.80906800
C	-2.13284900	-3.14551000	1.33787200
C	-0.11847200	-3.92325400	3.10939300
H	0.31762400	-1.82692600	3.28023600
C	-1.96062100	-4.48806300	1.66650600
H	-2.91287700	-2.86982200	0.64559900
C	-0.95412800	-4.88516100	2.54261800
H	0.65530200	-4.21840900	3.81140700
H	-2.62815500	-5.22756100	1.23541700
H	-0.83201700	-5.93320700	2.79673300

PD-B

C	0.79634800	-0.16875500	2.57814500
O	0.51973800	-0.87780200	3.51260100
O	1.95553700	0.50701600	2.50003000
C	2.91972700	0.17099800	3.49438800
H	3.16654100	-0.89163800	3.42461900
H	3.79433600	0.78065000	3.27339300
H	2.53266400	0.38643500	4.49220500
C	-0.14121400	0.11878600	1.42610700
C	0.29957400	-0.07173300	-0.05590100
C	-1.36441000	0.45631800	1.85298500
C	-0.52118100	-1.26673300	-0.75903800
H	-1.50075600	0.44810800	2.93418800
C	-0.92866000	0.76153200	-2.02332700
C	-1.54571900	-0.58518700	-1.68857700
H	-1.71527400	1.51660000	-2.12322400
H	-2.49406600	-0.45410400	-1.16843500
H	-1.73494100	-1.18731300	-2.57956700
N	-0.08233000	1.07805100	-0.88428500
C	-1.14300800	-2.27393600	0.20500900
O	-0.68840500	-2.60488900	1.26893400
O	-2.24138600	-2.81937800	-0.33350600
C	-2.79693500	-3.89812200	0.41735600
H	-2.05921200	-4.69625200	0.52272800
H	-3.09616000	-3.55732200	1.41131700
H	-3.66104300	-4.23935300	-0.15016000
C	0.39787100	-2.13356800	-1.63012700
O	0.61896400	-3.30253100	-1.44083900
O	0.90427000	-1.43383700	-2.65102700
C	1.95580600	-2.09357900	-3.36001400
H	1.58442800	-3.00554400	-3.83097200
H	2.29897400	-1.38064200	-4.10774900

H	2.76222700	-2.34073000	-2.66419100
H	-0.35089600	0.73357300	-2.95688600
C	-2.60298200	0.71709600	1.08282500
C	-2.80561200	1.87664400	0.33050800
C	-3.62861300	-0.23291800	1.16454200
C	-3.99398700	2.05216100	-0.37408900
H	-2.02800200	2.63417100	0.29184900
C	-4.81116800	-0.05990900	0.45243300
H	-3.47841100	-1.11957200	1.77628700
C	-4.99208500	1.08062300	-0.32756900
H	-4.13816400	2.95355800	-0.96260700
H	-5.59036400	-0.81409600	0.50646400
H	-5.91303000	1.21842900	-0.88558300
C	0.34416100	2.38791000	-0.69166700
C	1.10092700	2.78098000	0.42818500
C	0.01590300	3.38140600	-1.63710100
C	1.47262700	4.10737500	0.60199000
H	1.42234400	2.05064100	1.15658800
C	0.39659300	4.70593200	-1.44373600
H	-0.52163300	3.12529800	-2.54124000
C	1.12046100	5.08885800	-0.32140300
H	2.05449000	4.37079100	1.48059700
H	0.12304000	5.44180800	-2.19451100
H	1.41443500	6.12259600	-0.17462700
C	1.80592100	-0.41142800	-0.14090400
C	2.28434900	-1.54901000	0.52105200
C	2.68947000	0.33188300	-0.92085800
C	3.62613900	-1.90685200	0.43775700
H	1.59776200	-2.16546500	1.09908400
C	4.03499800	-0.02501900	-1.00037300
H	2.33179900	1.18976000	-1.47829500
C	4.51145900	-1.13860700	-0.31602300
H	3.97519800	-2.79418800	0.95725200
H	4.70987100	0.57397500	-1.60449300
H	5.55985200	-1.41404600	-0.37904000

Simplified Models

CP-trans

C	-0.78541100	-1.67184700	-0.58465300
O	-0.91395600	-2.28187000	-1.61123400
O	-0.59264800	-2.21035400	0.62199800
C	-0.26561400	-3.60190500	0.60304400
H	0.60158200	-3.74624000	-0.04337800
H	-0.02671800	-3.85760900	1.63389200
H	-1.11729900	-4.18330500	0.23620000
C	-0.79806700	-0.16381100	-0.48428800
C	-1.47640800	0.45017200	0.65139300
C	-0.02372600	0.49553800	-1.38257100
H	0.57930500	-0.14483000	-2.02545600
N	-2.60836100	-0.07230300	1.09461600
C	-3.18254700	0.21130800	2.41752500
H	-2.44159400	0.69494200	3.04922100
H	-4.06624900	0.84543400	2.31271800
H	-3.48395200	-0.73800800	2.86127900
C	-3.35691800	-1.01448900	0.29443200
C	-3.85685900	-0.60042100	-0.93575900
C	-3.55536300	-2.31383700	0.75551800
C	-4.55577600	-1.50751900	-1.72484100
H	-3.68564100	0.41712100	-1.27376700
C	-4.25323500	-3.21219900	-0.04240300
H	-3.11898400	-2.62987200	1.69779000
C	-4.75160600	-2.81158900	-1.28084200
H	-4.93880700	-1.19423500	-2.68991900
H	-4.39409500	-4.23241000	0.29862100
H	-5.28674000	-3.52078400	-1.90332000
C	-0.87713600	1.60393100	1.34263000
C	0.49925600	1.60520500	1.58991100
C	-1.66095800	2.72111000	1.67165600
C	1.08266300	2.72754400	2.17057000
H	1.10638500	0.74013500	1.34096100
C	-1.06542600	3.83998000	2.23064400
H	-2.72326100	2.72812600	1.43843600
C	0.30935500	3.84096300	2.48191400
H	2.15069400	2.72451800	2.35903400
H	-1.66407600	4.71575600	2.45864200
H	0.77472500	4.72015200	2.91688500
C	0.06294100	1.93670800	-1.62158000
C	1.30240500	2.49606900	-1.96468900
C	-1.07059800	2.75765300	-1.56854500
C	1.40238600	3.86074700	-2.20468200
H	2.17625000	1.85260400	-1.98150600
C	-0.96837800	4.11907100	-1.82395900
H	-2.04107500	2.31687300	-1.35163000
C	0.27143700	4.67319400	-2.13590300
H	2.36739700	4.29270900	-2.44944200
H	-1.85419200	4.74567300	-1.78964900
H	0.35375400	5.73718600	-2.33593900
O	2.71648600	0.34547700	-0.36498900
S	2.59208600	-1.06411000	0.05485100
O	2.13307400	-1.24220300	1.43864500
O	1.93722500	-1.93229600	-0.93743000
C	4.32571300	-1.65839000	0.07867100
F	5.05171100	-0.95017300	0.94412400
F	4.88038900	-1.54068900	-1.12708400
F	4.37495500	-2.94038100	0.44133000

TS-trans

C	1.09101800	-1.64013700	0.60427100
O	1.26960800	-2.12982500	1.69028200
O	1.19862600	-2.30734000	-0.55331200
C	1.41372700	-3.70945800	-0.39841700
H	0.57792600	-4.13833800	0.15739700
H	1.45483500	-4.11460800	-1.40876100
H	2.35073400	-3.89304900	0.13475600
C	0.65864200	-0.22359400	0.37634000
C	1.19327000	0.56554900	-0.67139500
C	-0.37380200	0.21961500	1.20627600
H	-0.63583400	-0.47822300	1.99836100
N	2.42533000	0.36804900	-1.15380400
C	2.89557500	0.95768400	-2.41277100

H	2.04764800	1.28517200	-3.01010400
H	3.55755400	1.80478600	-2.21072500
H	3.45544100	0.19792700	-2.96028500
C	3.41678700	-0.37864100	-0.42412600
C	3.81297400	0.08285500	0.82793700
C	3.98964700	-1.52067400	-0.97862500
C	4.78363100	-0.61498200	1.53923600
H	3.35185300	0.97787900	1.23530100
C	4.96330300	-2.20887800	-0.26361600
H	3.64151300	-1.88546500	-1.94017400
C	5.35891400	-1.75953400	0.99521300
H	5.08762200	-0.26306500	2.51926600
H	5.40416200	-3.10599700	-0.68603200
H	6.11282700	-2.30511500	1.55300700
C	0.39476400	1.66658800	-1.26335100
C	-0.82425400	1.38067900	-1.88066000
C	0.84985700	2.98612900	-1.15682100
C	-1.58616700	2.42892400	-2.39256500
H	-1.17618500	0.35490300	-1.95027600
C	0.06588700	4.02507900	-1.63769700
H	1.79043600	3.19297500	-0.65162400
C	-1.15160400	3.74420600	-2.26045000
H	-2.53206300	2.21050000	-2.87728400
H	0.39755700	5.05186600	-1.52189400
H	-1.76274800	4.55761700	-2.63967700
C	-0.59220500	1.66860200	1.50043000
C	-1.74224400	2.39594700	1.19237000
C	0.47046600	2.31050600	2.14987000
C	-1.80369300	3.75329500	1.49659800
H	-2.56275300	1.90372500	0.68757900
C	0.39382700	3.66200300	2.46961500
H	1.35563700	1.73761700	2.41426800
C	-0.74188700	4.39203900	2.13107300
H	-2.69369300	4.31518900	1.22978800
H	1.22392100	4.14287100	2.97828900
H	-0.80153900	5.45022800	2.36625600
O	-2.18352500	-0.19999700	0.38326400
S	-2.27250800	-1.59807800	-0.18120500
O	-2.14064800	-1.63356400	-1.63448600
O	-1.50992900	-2.57068500	0.59714900
C	-4.03394100	-1.99067400	0.15202100
F	-4.82507500	-1.11385000	-0.46047300
F	-4.27739500	-1.94731200	1.45745800
F	-4.31419900	-3.20928000	-0.29801200

IM-trans

C	1.07877700	-1.69648700	0.46057100
O	1.04167400	-2.08343800	1.60866000
O	1.68779000	-2.36327100	-0.52415800
C	2.43964500	-3.49663700	-0.08881600
H	1.77567500	-4.23905800	0.35824100
H	2.91888900	-3.89354400	-0.98256300
H	3.19004000	-3.18672200	0.64389900
C	0.43515800	-0.43282800	0.01076500
C	1.03462200	0.43936600	-0.84308100
C	-0.84244500	-0.13469500	0.77040000
H	-0.96061600	-0.92850300	1.51278200
N	2.36318600	0.34706400	-1.26127600
C	2.63519300	0.49735300	-2.68530100
H	1.89247800	1.16322300	-3.12434100
H	3.62853900	0.93047200	-2.82518200
H	2.58603500	-0.46909200	-3.20340000
C	3.39409400	-0.17720100	-0.45036500
C	3.41540500	0.09918700	0.92447000
C	4.44397400	-0.91465700	-1.00754500
C	4.44839600	-0.37733200	1.71891000
H	2.61668600	0.69252600	1.35999500
C	5.48529700	-1.37254200	-0.20317100
H	4.44211200	-1.15223700	-2.06530500
C	5.49290200	-1.11648800	1.16308600
H	4.44268500	-0.15674200	2.78180400
H	6.29015100	-1.94423000	-0.65543700
H	6.30168700	-1.48057900	1.78778100
C	0.33086400	1.66319800	-1.32389700
C	-0.83380100	1.59645600	-2.09072800
C	0.86568800	2.90958100	-0.97983500
C	-1.47475500	2.77004200	-2.48245600
H	-1.23805500	0.63146900	-2.37954100
C	0.21207100	4.07701900	-1.35306400
H	1.77936100	2.94863400	-0.39232200
C	-0.96181300	4.00759700	-2.10337500
H	-2.37719100	2.71292900	-3.08294000
H	0.61507200	5.04090600	-1.05849000
H	-1.47027700	4.91987100	-2.40044600
C	-0.85392800	1.19915500	1.48237900
C	-1.69854900	2.25057500	1.14168800
C	0.07146600	1.35627000	2.51796000
C	-1.59714100	3.46547200	1.81812400
H	-2.41429400	2.13086300	0.33563100
C	0.16871900	2.56875600	3.18992800
H	0.70769200	0.51489000	2.78899700
C	-0.66395500	3.63037400	2.83606100
H	-2.24872300	4.28736200	1.53725000
H	0.88910900	2.68421900	3.99391900
H	-0.58721200	4.57927500	3.35777000
O	-2.02688700	-0.17504400	-0.09813600
S	-2.48642300	-1.57945300	-0.68545900
O	-2.91415000	-1.37177700	-2.04583800
O	-1.56716500	-2.62710900	-0.30634400
C	-3.99731100	-1.79895100	0.34047100
F	-4.85172900	-0.82002700	0.09629700
F	-3.64962400	-1.77856000	1.61967800
F	-4.55208000	-2.96302600	0.04576000

IM-cis

C	-0.23513800	-1.87426000	1.67792900
O	-0.96844600	-2.00328700	2.63321000
O	0.43322900	-2.90342600	1.12589600
C	0.17865200	-4.16532100	1.74037200
H	-0.88264200	-4.40916000	1.65942000

H	0.78385600	-4.88771800	1.19456200
H	0.45986700	-4.14371800	2.79652600
C	-0.01999900	-0.60234100	0.94696200
C	1.19759100	-0.20473500	0.46957900
C	-1.26295200	0.24910600	0.86478200
H	-1.99291600	-0.18081500	1.55775800
N	2.40467600	-0.70308700	0.91314200
C	2.57279600	-1.34488800	2.20966500
H	1.78608600	-0.99849600	2.88395900
H	2.53344300	-2.43597300	2.13858200
H	3.54098400	-1.05277800	2.62323800
C	3.48029000	-0.85470700	-0.01273200
C	3.24909100	-1.48350100	-1.23637500
C	4.75980700	-0.39785700	0.30535100
C	4.29033200	-1.63548900	-2.14623800
H	2.25177100	-1.85563400	-1.45482300
C	5.80001700	-0.56799800	-0.60143100
H	4.93133400	0.10815500	1.25109900
C	5.56854300	-1.18291600	-1.83030500
H	4.10286300	-2.11972100	-3.09939300
H	6.79260800	-0.20603500	-0.35225300
H	6.38167700	-1.30759800	-2.53806000
C	1.31481200	0.93818000	-0.48072600
C	0.68494200	0.91547500	-1.72578300
C	2.04152600	2.06747300	-0.09067300
C	0.76361300	2.02438800	-2.56431700
H	0.13184500	0.03424100	-2.03612000
C	2.09997700	3.18033100	-0.91924100
H	2.52695800	2.07405800	0.88142200
C	1.45900900	3.15980600	-2.15769900
H	0.27649100	1.99952200	-3.53401800
H	2.64071400	4.06557000	-0.59949700
H	1.50770600	4.02825400	-2.80772700
C	-1.05891600	1.71274200	1.19696200
C	-1.34240900	2.74782700	0.31093300
C	-0.53586400	2.00667900	2.45876100
C	-1.07679700	4.06655900	0.67648800
H	-1.74103400	2.52445300	-0.67203300
C	-0.28054500	3.32271500	2.82317600
H	-0.32460800	1.19179100	3.14833100
C	-0.54380600	4.35835100	1.92715200
H	-1.28244000	4.86636500	-0.02844800
H	0.12518700	3.54098100	3.80634200
H	-0.33673400	5.38669700	2.20671700
O	-1.88105700	0.21236800	-0.47799600
S	-2.53913500	-1.13043900	-1.00413100
O	-2.20860200	-1.27091200	-2.40010200
O	-2.37476900	-2.20739800	-0.05504200
C	-4.29690100	-0.60021000	-0.90350800
F	-4.49990400	0.43434300	-1.70229500
F	-4.56635000	-0.25491600	0.34798100
F	-5.07468500	-1.60665000	-1.26721000

TS-cis

C	-0.42155300	-1.73443900	1.87426000
O	-1.00390300	-1.87280800	2.92008700
O	-0.09768900	-2.74855600	1.05389400
C	-0.68888200	-4.00027400	1.41399600
H	-1.77460900	-3.89014300	1.41202200
H	-0.36740500	-4.70745900	0.65115900
H	-0.34915900	-4.31356600	2.40438200
C	0.00636000	-0.41875900	1.30607200
C	1.22569300	-0.27367600	0.59819400
C	-0.93389000	0.61055700	1.41097600
H	-1.79596200	0.36921600	2.02833800
N	2.30954500	-0.99813900	0.89943900
C	2.43849300	-1.78182600	2.12977800
H	1.78731800	-1.36496600	2.89919200
H	2.17555900	-2.82671600	1.94825000
H	3.47683200	-1.72186200	2.45987800
C	3.35148800	-1.21246700	-0.07182200
C	3.02830400	-1.85357300	-1.26488200
C	4.66021300	-0.82947400	0.20683500
C	4.03039900	-2.09405900	-2.19929800
H	1.99997800	-2.15536300	-1.44431300
C	5.65615800	-1.07969700	-0.73123200
H	4.89015700	-0.32304200	1.14018300
C	5.34241400	-1.70922900	-1.93405900
H	3.78380200	-2.58799700	-3.13329200
H	6.67698500	-0.77540600	-0.52459000
H	6.12190000	-1.90032000	-2.66432300
C	1.36340600	0.73112500	-0.48500200
C	0.50112000	0.69808100	-1.58195800
C	2.32208500	1.74326800	-0.36422300
C	0.60216600	1.68708700	-2.55673800
H	-0.24347600	-0.08717700	-1.67145800
C	2.38917300	2.74558400	-1.32109600
H	2.97407300	1.76602400	0.50478800
C	1.53057000	2.71475500	-2.42086500
H	-0.06365500	1.65863100	-3.41307900
H	3.10606900	3.55259500	-1.20927700
H	1.58701600	3.49769900	-3.17107200
C	-0.53002500	2.04696600	1.34735500
C	-1.01016500	2.97979000	0.42697000
C	0.40084200	2.45370200	2.31284200
C	-0.53508000	4.28793300	0.45699800
H	-1.71999500	2.66596200	-0.32665900
C	0.85141500	3.76875600	2.35241200
H	0.76984100	1.73142300	3.03619100
C	0.39241200	4.68890000	1.41472900
H	-0.89524700	4.99855900	-0.28061700
H	1.56757100	4.06910700	3.11126100
H	0.75308100	5.71273500	1.43176200
O	-2.20921900	0.56793300	-0.16914400
S	-2.70675100	-0.81041500	-0.53395100
O	-2.05417200	-1.35802500	-1.71931900
O	-2.84793200	-1.68580500	0.62725000
C	-4.42254400	-0.42879900	-1.06060600
F	-4.41218400	0.43549600	-2.07144300
F	-5.10660300	0.09997800	-0.05195500
F	-5.02783500	-1.54409900	-1.45454800

CP-cis

C	0.54903400	-0.85218400	-2.41593900
O	1.15078100	-0.67765300	-3.44457100
O	0.37305200	-2.03647600	-1.82994200
C	1.17517900	-3.09086900	-2.37214400
H	2.22458600	-2.80797000	-2.28342100
H	0.95275700	-3.96484900	-1.76221700
H	0.91388900	-3.26594400	-3.41903900
C	-0.10786800	0.27998500	-1.65321700
C	-1.25403100	-0.00190200	-0.80178000
C	0.60061900	1.44290600	-1.68199800
H	1.57418400	1.36071500	-2.16472300
N	-2.08678700	-0.98424000	-1.12369400
C	-2.26708000	-1.49343500	-2.48697300
H	-1.76947000	-0.83812600	-3.20112200
H	-1.87399200	-2.50753700	-2.56145300
H	-3.33886300	-1.49706400	-2.69833800
C	-2.72028500	-1.77148600	-0.09312200
C	-1.89390900	-2.30982400	0.89338700
C	-4.08474600	-2.04198800	-0.13533200
C	-2.46803900	-3.11629700	1.87081300
H	-0.82486800	-2.09541100	0.88128900
C	-4.64035000	-2.85411600	0.84763400
H	-4.71128300	-1.60732800	-0.90896000
C	-3.83410500	-3.38940400	1.85022800
H	-1.83764200	-3.53806400	2.64648900
H	-5.70471900	-3.06414800	0.83119900
H	-4.27228600	-4.02293600	2.61458800
C	-1.55629200	0.76690800	0.42407000
C	-0.55723300	1.23792800	1.28111800
C	-2.89371000	1.14072000	0.63663100
C	-0.90243000	2.07213500	2.33841800
H	0.47769900	0.96038500	1.12148700
C	-3.22592100	1.98300200	1.68579600
H	-3.66512200	0.79909300	-0.04739100
C	-2.22705100	2.44893300	2.54123500
H	-0.12147000	2.43388900	2.99876800
H	-4.25842200	2.28283100	1.83252800
H	-2.48448300	3.11048900	3.36287300
C	0.24694200	2.76570500	-1.17855800
C	1.25412900	3.53569400	-0.58048000
C	-1.05235600	3.27932000	-1.28191700
C	0.94525600	4.78045400	-0.04586000
H	2.25012100	3.11376000	-0.48493500
C	-1.35084100	4.53064600	-0.76136200
H	-1.82026900	2.69600800	-1.78278100
C	-0.35376600	5.27779600	-0.13422800
H	1.71987800	5.36422300	0.44065700
H	-2.35821200	4.92568200	-0.84524300
H	-0.58927500	6.25440700	0.27759700
O	2.52958100	0.91177200	0.58444200
S	2.48353700	-0.54179000	0.33496300
O	1.25938700	-1.19558400	0.83308500
O	2.89496500	-0.93639200	-1.01799000
C	3.80098700	-1.21518900	1.41595000
F	3.56146500	-0.90859000	2.69084700
F	4.98651900	-0.71439000	1.07763700
F	3.85571000	-2.54276900	1.30753700
